# New insights into the genetic diversity of the stone crayfish: taxonomic and conservation implications

**DOI:** 10.1186/s12862-020-01709-1

**Published:** 2020-11-06

**Authors:** Leona Lovrenčić, Lena Bonassin, Ljudevit Luka Boštjančić, Martina Podnar, Mišel Jelić, Göran Klobučar, Martina Jaklič, Valentina Slavevska-Stamenković, Jelena Hinić, Ivana Maguire

**Affiliations:** 1 Division of Zoology, Department of Biology, Faculty of Science, University of Zagreb, Rooseveltov trg 6, 10000 Zagreb, Croatia; 2 Croatian Natural History Museum, Demetrova 1, 10000 Zagreb, Croatia; 3 Department of Natural Sciences, Varaždin City Museum, Franjevački trg 10, 42000 Varaždin, Croatia; 4 Center for Clinical Research, University Medical Centre Ljubljana, Zaloška 2, 1000 Ljubljana, Slovenia; 5 Department of Invertebrates and Animal Ecology, Faculty of Natural Sciences and Mathematics, University “St. Cyril and Methodius”, Arhimedova 3, 1000 Skopje, Republic of North Macedonia

**Keywords:** *Austropotamobius torrentium*, Species delimitation, Species validation, MOTU, ESU, Phylogeographic patterns, nuDNA, mtDNA, Evolutionary history

## Abstract

**Background:**

*Austropotamobius torrentium* is a freshwater crayfish species native to central and south-eastern Europe, with an intricate evolutionary history and the highest genetic diversity recorded in the northern-central Dinarides (NCD). Its populations are facing declines, both in number and size across its entire range. By extanding current knowledge on the genetic diversity of this species, we aim to assist conservation programmes. Multigene phylogenetic analyses were performed using different divergence time estimates based on mitochondrial and, for the first time, nuclear DNA markers on the largest data set analysed so far. In order to reassess taxonomic relationships within this species we applied several species delimitation methods and studied the meristic characters with the intention of finding features that would clearly separate stone crayfish belonging to different phylogroups.

**Results:**

Our results confirmed the existence of high genetic diversity within *A. torrentium*, maintained in divergent phylogroups which have their own evolutionary dynamics. A new phylogroup in the Kordun region belonging to NCD has also been discovered. Due to the incongruence between implemented species delimitation approaches and the lack of any morphological characters conserved within lineages, we are of the opinion that phylogroups recovered on mitochondrial and nuclear DNA are cryptic subspecies and distinct evolutionary significant units.

**Conclusions:**

Geographically and genetically isolated phylogroups represent the evolutionary legacy of *A. torrentium* and are highly relevant for conservation due to their evolutionary distinctiveness and restricted distribution.

## Background

**Fig. 1:**
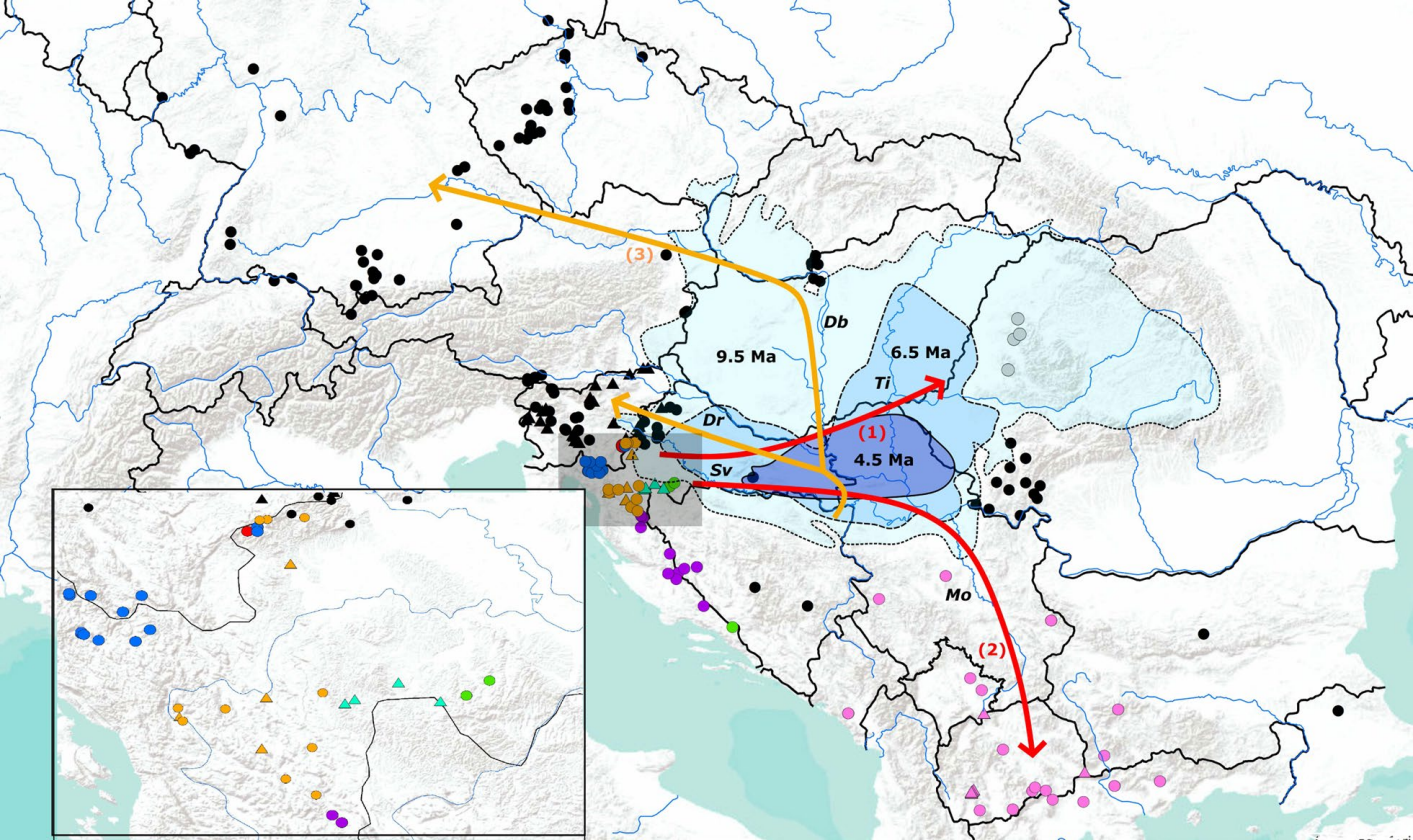
Geographical distribution of different *A. torrentium* mtDNA phylogroups in Europe produced in ArcGIS 10.3 program package and finished in the program Inscape 1.0 by authors of this study. Symbols used on the map: dots represent samples from previous research [3–5], and triangles samples from this study. Colours depict mitochondrial phylogroups: black—central and south-eastern Europe (CSE), blue—Gorski Kotar (GK), purple—Lika and Dalmatia (LD), orange—Žumberak, Plitvice and Bjelolasica (ŽPB), pink—southern Balkans (SB), green—Banovina (BAN), red—Zeleni Vir (ZV), gray—Apuseni Mountain (APU) and turquoise blue—Kordun (KOR), new phylogroup discovered in the present study. River systems abbreviations: *Db* Danube, *Dr* Drava, *Sv* Sava, *Ti* Tisza, *Mo* Morava. Also shown: the extent of the Lake Pannon at 9.5 Ma, 6.5 Ma and 4.5 Ma (adapted according to Magyar et al. [76]) and shaded in blue. Possible pre-glacial and post-glacial dispersal routes are indicated; red arrows indicate: (1) possible colonisation of the Apuseni Mountains through delta systems of paleo-Danube and paleo-Tisza on the northern shelf margin of the Lake Pannon and (2) colonization of southern Balkan after formation of the freshwater Danube drainage system. Orange arrows (3) indicate post-glacial recolonisation of northern part of *A. torrentium* areal through leading edge expansion of CSE phylogroup (adapted according to Klobučar et al. [4]). Shaded gray area in the main map is enlarged in the bottom left corner

The stone crayfish (*Austropotamobius torrentium* (Schrank, 1803)) is an indigenous European crayfish species (ICS) [1]. It is the smallest of all European ICS and is considered a keystone species in freshwater ecosystems [2]. The stone crayfish is a cold-adapted species active at water temperatures > 5 °C with a mean annual water temperature that does not exceed 10 °C [2]. It inhabits smaller pristine waterbodies at high altitude in central and south-eastern Europe (Fig. 1) that are related to karstic formations [1, 2]. The species exhibits high genetic diversity represented by the eight distinct mtDNA lineages/phylogroups discovered so far [3–5].

Lately, studies have shown that populations of the stone crayfish, as well as those of other ICS, are declining [1, 6]. They are threatened by habitat deterioration [2], water quality decline [7], climate change [6, 8], the presence/spreading of non-indigenous invasive American crayfish species and their pathogens, e.g., *Aphanomyces astaci* causative agent of the disease crayfish plague [9, 10]. ICS are offered differing levels of protection under international and national laws, with the stone crayfish listed in the Annexes II and V of the EU Habitats Directive [11]. The conservation status of *A. torrentium* remains unresolved as it noted as being “data deficient” on the global IUCN Red List [12], whilst in Croatia it is classed as vulnerable [13].

The maintenance of genetic diversity is considered fundamental to modern conservation efforts, as it is essential for securing the evolutionary potential and long-term survival of a species [14]. In order to protect vulnerable species adequate conservation plans are urgently needed on a global scale which requires sound knowledge of both the morphologic and genetic diversity of this species in addition to the identification of evolutionary independent lineages within the species [2, 15, 16].

The first morphological studies aimed to distinguish between different populations of the stone crayfish [17] which resulted in the identification of four subspecies: *Austropotamobius torrentium torrentium* [17], *A. t. macedonicus* [18], *A. t. dalmatinus* [18] and *A. t. danubicus* [19, 20]. Later, studies based mainly on meristic characteristics confirmed previously described subspecies [20, 21]. Recently, Maguire et al. [22] discovered differences among distinct populations (representing different mtDNA phylogroups defined by preciding genetic analyses [4]) of the stone crayfish in a small geographical region in Croatia. This was achieved by analysing a number of individual morphometric and meristic characteristics with these findings corroborated by a large scale geometric morphometric analyses applied to stone crayfish populations for the first time [23]. Even though these studies confirmed morphological delineation between phylogroups (the cephalon shape being pertinent), it was shown that morphological variation within phylogroups is also present [22, 23]. Freshwater crayfish are known to exhibit high intraspecific morphological variation and plasticity reflecting environmental influence and/or genetic background [22, 24, 25]. Hence, it is hard to find unique and unambiguous morphological character specific only for one phylogroup that would be suitable to clearly distinguish between phylogroups thus resolving the taxonomic status of *A. torrentium* phylogroups in addition to describing a new species.

Until now, molecular phylogenetic studies of *A. torrentium* were based on the analyses of mitochondrial genes for cytochrome *c* oxidase subunit I (*COI*) and *16S* ribosomal RNA (*16S* rRNA) [3–5, 26, 27]. Trontelj et al*.* [3] discovered three highly divergent mtDNA phylogroups: one distributed in the southern part of the Balkan Peninsula, another in a small area on the border between Slovenia and Croatia, and the third that encompasses the rest of Europe. This finding indicated that the stone crayfish should be considered a species complex. Later, Klobučar et al. [4] confirmed Trontelj et al. [3] findings, and discovered the existence of four additional phylogroups, with the highest genetic diversity found in the Dinaric region of Croatia. The phylogroups were named after geographical areas of their distribution: central and south-eastern Europe (CSE), southern Balkans (SB), Banovina (BAN), Gorski Kotar (GK), Lika and Dalmatia (LD), Zeleni Vir (ZV), Žumberak, Plitvice and Bjelolasica (ŽPB), with the five latter situated in the north and central Dinarides (NCD). Recently, Pârvulescu et al. [5] discovered the existence of a new phylogroup, endemic to the Romanian Apuseni Mountain region (APU). Combining the molecular mtDNA analyses with morphological data, the APU phylogroup was proposed as a new species *Austropotamobius bihariensis* [28].

Species delimitation requires integrative taxonomic approach that combines molecular, morphological, ecological, and geographical data to build species hypotheses [29, 30]. This approach enables taxonomy to go beyond naming the species and assists in understanding the processes that shape the species [31, 32].

Even though mitochondrial genes (*COI* and *16S* rRNA) are appropriate for resolving taxonomic relationships between genera and species [33–35], they show some drawbacks in species delimitations (e.g., higher failure rate at proposing species delimitation hypothesis compared to nuclear markers) [31, 36, 37]. Therefore, the need for a nuclear marker that can be used in the reconstruction of genetic relationships as well as in the species delimitation has been recognised [38, 39]. Yao et al. [40] proposed the second internal transcribed spacer (*ITS2*) as a nuclear marker that is complementary to mitochondrial *COI* and *16S* rRNA, and is suitable in studying relationships of lower taxonomic categories (e.g. genera, species) [41] as well as for species delimitation [42].

In order to extand current knowledge about the stone crayfish diversity and provide baseline for conservation programs, the aims of our study were:

- to update phylogenetic findings based on the largest dataset used so far that includes new samples from previously unstudied stone crayfish populations from Croatia, Slovenia and Republic of North Macedonia
- to test if *ITS2* is a good nuclear marker for phylogenetic inference on *A. torrentium* and verify phylogenetic congruence between mitochondrial and nuclear DNA markers
- to evaluate alternative scenarios in the background of the currently observed distribution, genetic variability and phylogeographic patterns via varied molecular clock calibrations
- to apply species delimitation methods aiming to identify Molecular Operational Taxonomic Units (MOTUs) within *A. torrentium,* and to reassess their taxonomic status
- to study meristic characteristics on a large data set in order to find reliable character/characters that will clearly and undoubtedly distinguish MOTUs
- to give new perspectives in *A. torrentium* conservation programs through the identification of Evolutionary Significant Units (ESUs).

## Results

### Sequence data

We obtained a total of 153 (58 new) *COI* and 72 (24 new) *16S* rRNA unique haplotypes. The concatenated *COI*/*16S* rRNA data set included 151 (78 new) haplotype combinations (Additional file 1). Analyses of *COI* gene revealed 166 (27.95%) variable sites, of which 141 (23.74%) were parsimony informative, while 65 (13.59%) sites were variable in *16S* rRNA sequences, with 51 (10.27%) of them being parsimony informative. Obtained *ITS2* sequences showed only 27 (2.45%) variable sites, and 20 (1.81%) were parsimony informative. Analysis of the *ITS2* sequences using FastGap revealed gapmatrix with 26 (2.35%) gap sites, 13 being informative.

### Phylogenetic reconstruction

**Fig. 2:**
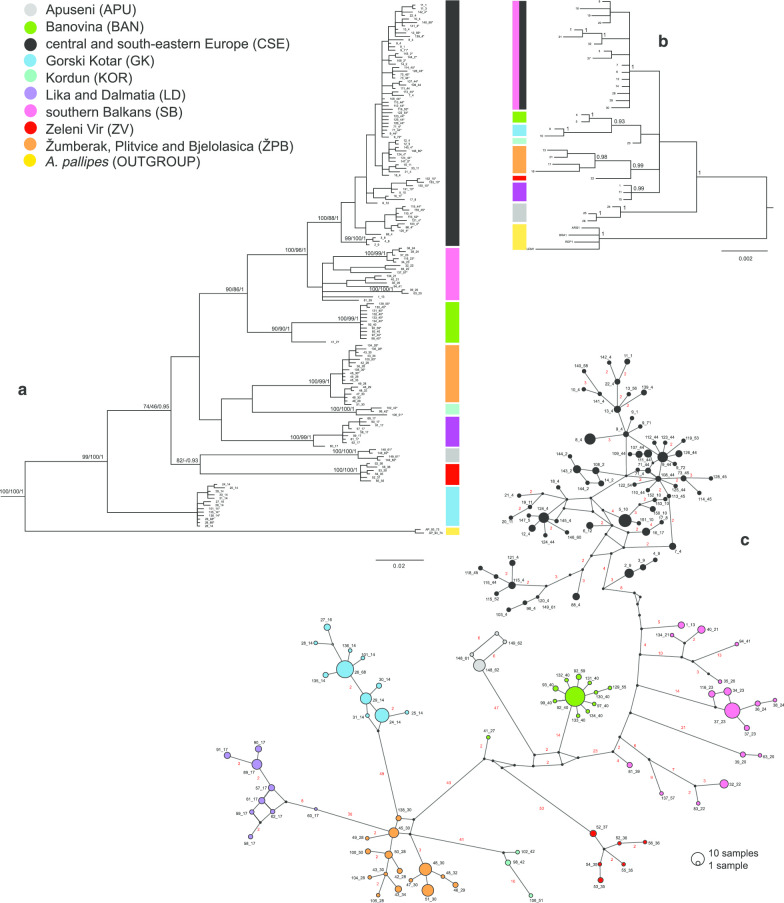
Phylogenetic recontruction for *A. torrentium*. **a** Phylogram inferred from concatenated *COI/16S* rRNA (new haplotypes obtained in this study are marked with an asterisk) and **b** phylogram inferred from *ITS2* haplotypes depicting the phylogenetic relationships within *A. torrentium*. Numbers at the nodes indicate maximum likelihood and maximum parsimony nonparametric bootstrap support values and Bayesian posterior probabilities, respectively. **c** Median joining (MJ) network for concatenated *COI*/*16S* rRNA. Numbers of mutational steps are given in red above branches except when it equals one. The size of the circle is proportional to the frequencies of the haplotype. The black dots indicate extinct ancestral or unsampled haplotypes. Phylogroups are represented by different colour: black—central and south-eastern Europe (CSE), blue—Gorski Kotar (GK), purple—Lika and Dalmatia (LD), orange—Žumberak, Plitvice and Bjelolasica (ŽPB), pink—southern Balkans (SB), green—Banovina (BAN), red—Zeleni Vir (ZV), gray—Apuseni Mountain (APU) and turquoise blue—Kordun (KOR)

All implemented criteria of phylogenetic reconstruction (BA, MP and ML) yielded mostly congruent topologies for *COI*/*16S* rRNA concatenated data set (Fig. 2a). The new phylogroup, belonging to the Kordun region (part of NCD) was discovered, while majority of the newly obtained sequences nested within the eight previously reported phylogroups [4, 5]. Moreover, phylogroups belonging to the NCD region (ZV, GK, ŽPB, LD, BAN, KOR) and APU appeared as monophyletic clades, well supported by bootstrap values and Bayesian posterior probabilities (Fig. 2a). The ‘Southern Balkans’ (SB) phylogroup was not supported as monophyletic; it comprised four sub-clades and two individual haplotypes represented in a basal polytomy with the monophyletic CSE clade. Numerous sub-clades also existed within well-supported monophyletic CSE phylogroup.

The Bayesian inference of phylogeny based on the nuclear gene *ITS2* yielded a tree topology with seven well supported phylogroups and the KOR lineage (Fig. 2b). Unlike in mtDNA phylogeny, CSE haplotypes have not formed separate monophyletic clade, but rather combined with SB haplotypes in a well supported clade. Phylogenetic relations among groups were not resolved with the majority of them form polytomy within *A. torrentium*, with the exception of well-supported separation of APU phylogroup (Fig. 2b).

In general, the common feature of the phylogenetic recontructions of both datasets was that phylogroups were well supported in the phylogenetic trees, but the relationship among them was unresolved, showing weak support for deeper nodes.

### Phylogeographic analysis and genetic diversity

A median-joining (MJ) network for concatenated *COI*/*16S* rRNA data set was used to visualise haplotype relatedness and haplotype distribution within *A. torrentium* (Fig. 2c). All nine phylogroups were highly divergent and separated by large numbers of mutational steps. The newly discovered KOR phylogroup was 42 mutational steps distant from closely related ŽPB phylogroup. The CSE phylogroup showed a complex structure consisting of large number of closely related haplotypes with a broad geographical distribution, separated by a small number of mutational steps. The SB phylogroup comprised six subclades separated by a large number of mutational steps. The SB and CSE phylogroups showed the smallest between-group number of mutational steps, whilst the ZV phylogroup showed the largest number of mutational steps when related to its closest neighbouring phylogroup BAN. Further, contrary to the relations in the phylogenetic tree, the APU phylogroup was closest to the BAN and not to the ZV phylogroup.

The results of TCS network analysis, based on the *COI* data set (used also as species delimitation approach; Additional file 2), were concordant with MJ results. The TCS network revealed the existence of 18 MOTUs with CSE, GK, ZV, LD, KOR, APU and ŽPB phylogroup each representing one MOTU. The SB phylogroup was split into nine separated MOTUs. The BAN phylogroup was split into two MOTUs; haplotype 41 formed the first one, and the second contained all other BAN haplotypes.

The obtained values of uncorrected sequence divergences (p-distances) and patristic distances within and between phylogroups for *COI, 16S* rRNA and *ITS2* are shown in Additional file 3. The obtained values of genetic distances for all genes were calculated using p-distances and K2P distances were congruent. The p-distances between phylogroups ranged from 4.98 to 9.62% for *COI*, and from 0.00 to 5.05% for *16S* rRNA gene. The highest values of genetic distances were observed when ZV, GK and APU clades were compared with other phylogroups, for both *16S* rRNA and *COI* markers. The range of p-distances within phylogroups for the *COI* gene was between 0.17 and 5.33%, and between 0.00 and 2.75% for *16S* rRNA gene. The values of p-distances for the *ITS2* gene, which showed less genetic variation than mitochondrial genes, were mostly congruent to the results obtained for mitochondrial genes, ranging from 0.00 to 0.79% between groups, and from 0.00 and 0.29% within groups (Additional file 3). Patristic distances between the phylogroups indicated various molecular divergence between several phylogroup pairs with values ranging from 0.08 to 0.22.

### Time of divergence

**Fig. 3:**
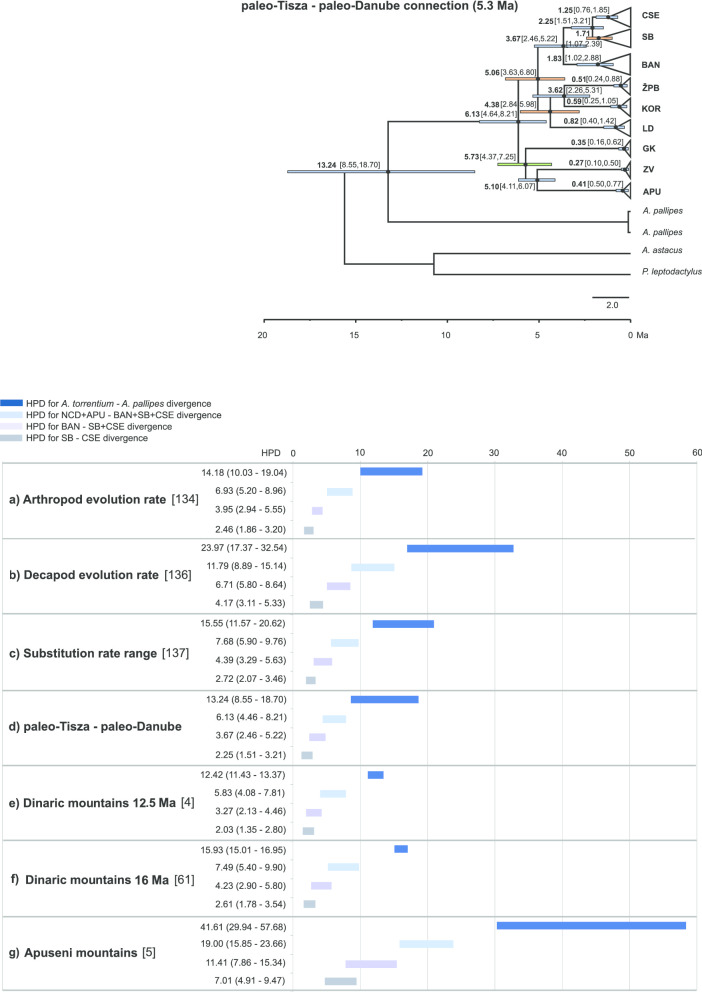
Chronogram of 95% highest posterior density intervals (HPD) of divergence time estimates (in Ma) obtained with the mean values in brackets **a** using arthropod evolutionary rate [134, 135], **b** using decapod evolutionary rate [136], **c** using mid-points of a uniform distribution [137], **d** using geological calibration based on the connection of paleo-Tisza–paleo-Danube river systems **e** and **g** using geological calibration based on the uplift of the Dinaric Mountains [4, 61], **f** using geological event based on the separation of the Tisza–Dacia microplate from Dinarides [5]. Different colours denote the HPD of distinct lineages: dark blues—split of *A. pallipes* and *A. torrentium*; light blue—split of NCD (north and central Dinaric phylogroups = ZV, GK, LD, KOR, ŽPB) + APU from the BAN, SB and CSE phylogroups; purple—split of BAN from CSE + SB phylogroups; grey—split of CSE and SB phylogroups. In the upper right corner BEAST estimates of divergence times for *A. torrentium* based on the paleo-Danube–paleo-Tisza geological calibration is given; maximum clade credibility tree based on concatenated sequence. Horizontal node bars depict the 95% HPD intervals and are coloured according to posterior probability support (blue bars—posterior probabilities > 0.95; orange bars—posterior probabilities 0.50–0.95, green bars—posterior probabilities < 0.50. *APU *Apuseni, *ZV* Zeleni Vir, *GK* Gorski Kotar, *LD* Lika and Dalmatia, *KOR* Kordun, *ŽPB* Žumberak, Plitvice and Bjelolasica, *BAN* Banovina, SB southern Balkans, *CSE* central and southeastern Europe. *Austropotamobius pallipes*, *Astacus astacus* and *Pontastacus leptodactylus* were used as outgroups

Divergence time estimates based on a mitochondrial data set using three molecular clock and four geological calibrations are presented in Fig. 3 (for details see Additional file 4). The results of divergence time approximations overlapped, with the mean values of three molecular calibration approaches as follows: (a) ~ 17.90 Ma for the split between *A. pallipes* and *A. torrentium*, (b) ~ 8.80 Ma for the split between populations belonging to the NCD + APU from BAN + SB + CSE phylogroups, (c) ~ 5.01 Ma for the split of SB + CSE phylogroups from BAN phylogroup, and (d) ~ 3.12 Ma for the split between SB and CSE phylogroups.

Geological calibration points showed a wider range of different divergence times estimates. The Tisza–Dacia microplate tectonic displacement that, according to Pârvulescu et al. [5], occurred ~ 16 Ma, gave the largest intervals of possible divergence times, and was not consistent with other geological and molecular calibrations. The results of new geological calibration point used in this research (Fig. 3), based on the contact between the paleo-Tisza and paleo-Danube river systems [43], accompanied by the process of desalination of the Lake Pannon, indicated that this dispersion route could have enabled colonisation of the species north-east distribution range including the Apuseni region. Estimates of this calibration approach yielded results consistent with molecular and geological calibrations based on the intense Dinarides uplift. The median values for the key points in *A. torrentium* evolution based on this geological calibration were: (a) 13.24 Ma (HPD 18.70–8.55 Ma) for the split of *A. pallipes* and *A. torrentium*, (b) 6.13 Ma (HPD 8.21–4.46 Ma) for the split of NCD + APU from BAN + SB + CSE phylogroups, (c) 3.67 Ma (HPD 5.22–2.46) for the split between BAN and CSE + SB phylogroups and (d) 2.25 Ma (HPD 3.21–1.51 Ma) for the split between CSE and SB phylogroups.

### Species delimitation and validation

**Fig. 4:**
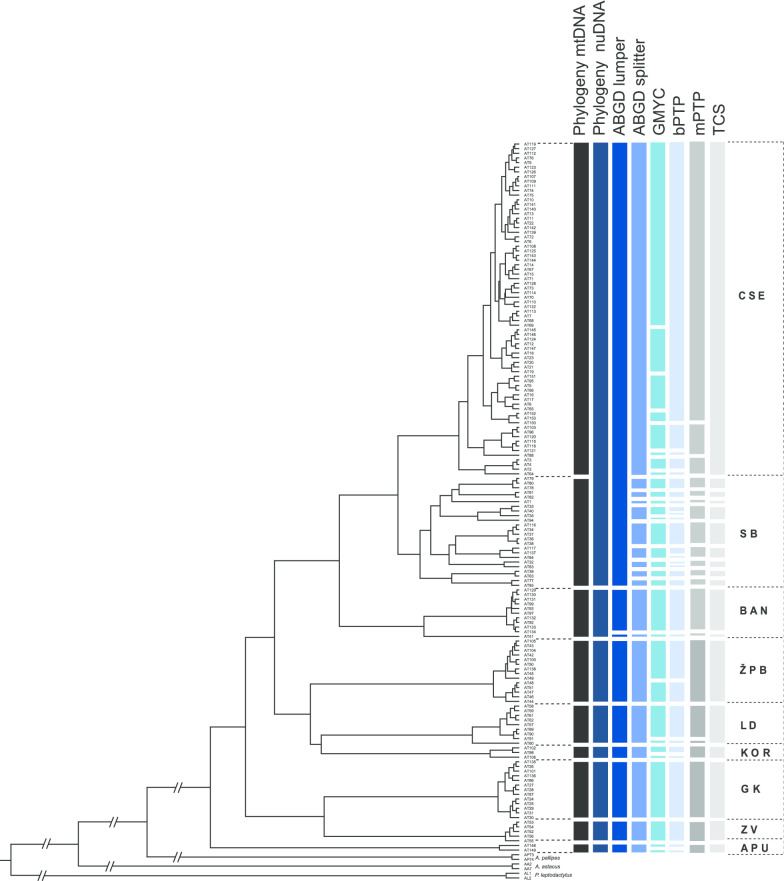
Species delimitation results visualised as bars on an ultrametric Bayesian maximum clade credibility tree of *A. torrentium COI* gene. Next to the tree phylogroups obtained according to reconstruction of mtDNA and nuDNA are presented. Then follows two partitions of Automatic Barcode Gap Discovery (lumper and splitter) (ABGD [101]), General Mixed Yule Coalescent (GMYC [102]); Bayesian implementation of the Poisson Tree Processes (bPTP [140]); Multi-rate Poisson Tree Processes (mPTP [141]), and Templeton, Crandall and Sing method (TCS [129]). Also, in the last column phylogroups’ abbreviations are given: *ZV* Zeleni Vir, *GK* Gorski Kotar, *ŽPB* Žumberak, Plitvice and Bjelolasica, *LD* Lika and Dalmatia, *BAN* Banovina, *SB* southern Balkans, *CSE* central and southeastern Europe, *APU* Apuseni. As outgroups *Austropotamobius pallipes*, *Astacus astacus* and *Pontastacus leptodactylus* were used

Species delimitation analyses (ABGD, GMYC, bPTP, mPTP, TCS) for mtDNA (*COI*) confirmed the existence of different *A. torrentium* MOTUs (Fig. 4, Additional file 5). The number of supported groups varied depending on the applied method. In the ABGD analysis for the majority of prior intraspecific divergence values (P), initial partitioning identified nine MOTUs (ABGD lumper approach), while the results from recursive partitioning singled out the existence of 18 MOTUs (ABGD splitter approach). The ABGD lumper, as the most conservative approach, recognised six phylogroups as a single MOTU: APU, GK, KOR, LD, ZV, ŽPB, while BAN phylogroup was split into two MOTUs. CSE and SB phylogroups were lumped into one MOTU. The ABGD splitter revealed nine MOTUs in the SB phylogroup, while haplotypes belonging to CSE phylogroup were recovered as a single MOTU. Delimitation results from ABGD splitter were consistent with the results obtained by TCS method. The mPTP method delimited 21 putative MOTUs that were mostly congruent with the results from ABGD splitter and TCS*.* The bPTP recognised between 26 and 45 MOTUs, 9 with Bayesian support values over 0.95. The GMYC *single threshold* approach identified 22 ML clusters (confidence interval: 19–36) and 29 entities (confidence interval: 25–53), but most of them lacked statistical support. Overall, PTP and GMYC yielded unrealistically high number of MOTUs, and relying only on the supported groups, the number of recognised groups was lower. Nested sampling analysis yielded marginal likelihood estimations ranging from − 4195 to − 4539 (Additional file 6). The model receiving the highest marginal likelihood score was GMYC, and calculated Bayes factor values showed decisive support for species tree topology associated with this species delimitation.

**Fig. 5:**
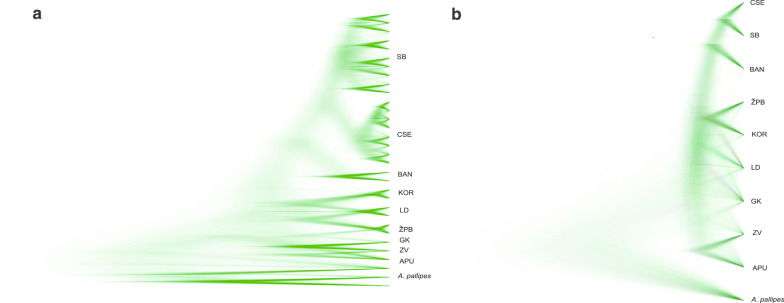
Species tree of *A. torrentium* inferred with *BEAST and visualised with DENSITREE, based on GMYC species delimitation model (**a**) and multilocus species delimitation (**b**). Next to the trees phylogroups’ abbreviations are given: *ZV* Zeleni Vir, *GK* Gorski Kotar, *ŽPB* Žumberak, Plitvice and Bjelolasica, *LD* Lika and Dalmatia, *BAN* Banovina, *SB* southern Balkans, *CSE* central and southeastern Europe, *APU* Apuseni. *Austropotamobius pallipes* was used as an outgroup

Single-locus species tree (*COI*) based on GMYC and multi-locus species tree (*COI* + *16S* rRNA + *ITS2*) based on the phylogeny showed a pattern of divergence between phylogroups; all phylogroups formed own monophyletic clades (Fig. 5). Species trees were congruent, showing the pattern of high genetic diversity, with no clear separation of genetic clusters (phylogroups).

### Meristics

Within-phylogroup variation in the number of spines on the ventral side of the merus of the third maxilliped was apparent, while significant difference between studied phylogroups was obtained (H (7, N = 732) = 112.94, P < 0.001), with crayfish from ZV possessing more spines compared to crayfish from other phylogroups, except KOR (Additional file 7).

Presence and pronunciation of rostral crista was inconsistent, and differed among phylogroups (χ^2^_21, N = 735_ = 491.58, P < 0.01); some of the crayfish from KOR, CSE and LD did not have rostral crista; in the rest of phylogroups rostral crista were present, and variation in the level of pronunciation exists. Crayfish from GK did not have weak crista, and in the SB phylogroup we did not record any crayfish with strong crista (Additional file 7).

In the studied phylogroups all three types of denticulation on the lower surface of antennal exopodite (smooth = no denticulation, tubercles, spines) were recorded, with phylogroups differed in the percentage of different type of denticulation (χ^2^_14, N = 735_ = 176.22, P < 0.01) (Additional file 7).

The “shape” of the tip of the endopodit of the first gonopod differed among males from different phylogroups (χ^2^_7, N = 414_ = 151.67, P < 0.01), with a small percentage of males from CSE, ŽPB and KOR possessing a 1st gonopod tip measuring half the length of the gonopod (Additional file 7). The length of the second gonopod tip did not differ among males from different phylogroups (χ^2^_7, N = 414_ = 7, P = 0.42), with all males, but a small percentage from ŽPB, posses the tip of the 2nd gonopod that was a third of the length of the gonopod (Additional file 7). The length of the exopodite of the second gonopod differed among males from different phylogroups (χ^2^_7, N = 414_ = 209.82, P < 0.01). Variation within phylogroups was evident, with the exception of males from ZV who all possessed an exopodite that was half the length of the second gonopod (Additional file 7).

## Discussion

### Phylogenetic structure, genetic diversity and phylogeographic analysis

This study confirmed the complexity of *A. torrentium*’s phylogenetic structure which consists of nine highly divergent and genetically diverse phylogroups [3–5]. An important discovery of this study was the establishment of novel haplotypes distributed in the Kordun region (part of NCD) forming a new Kordun phylogroup (KOR) (Fig. 2a, c). This is the result of the comprehensive sampling of a previously poorly studied region and indicates that future studies could potentially reveal more diversity within stone crayfish. All phylogroups were well supported as deeply divergent monophyletic clades, with the exception of the SB phylogroup that shows a paraphyletic relationship towards CSE phylogroup on both a mitochondrial and nuclear phylogenetic reconstructions*.* Even though the phylogroups were highly supported, their phylogenetic relationship is best described as unresolved polytomy (Fig. 2a, b). This lack of resolution could have emerged from a rapid and simultaneous divergence of the phylogroups [4, 44, 45].

For both mitochondrial genes, ranges of genetic distances between and within the phylogroups (Additional file 3) were in accordance with previously reported in Trontelj et al. [3], Klobučar et al. [4], Petrusek et al. [27] and Berger et al. [46]. Some of the observed ranges of the *COI* genetic distances between phylogroups were within the range of genetic distances found between *Astacus* species [3, 47], *Austropotamobius* species [3, 48] or Australian Parastacidae crayfish [49]. The lowest value of sequence divergence calculated between SB and CSE demonstrate their genetic similarity. Namely, ancestors of SB phylogroup went through a southern expansion [4, this study], presumably through paleo-Morava, right tributary of the paleo-Danube. This idea is supported by the fact that the oldest SB clades are distributed nowadays in Serbia’s Morava tributaries (Fig. 1). Populations of SB sub-clades were probably isolated during glaciations in the numerous micro-refugia in the southern part of the Balkan Peninsula and did not come into the secondary contact post-glacially which resulted in high genetic distances among them, which is similar to the findings of Laggis et al. [50] for the noble crayfish (*Astacus astacus*) and Economidis and Banarescu [51] for freshwater fishes. Further, the CSE phylogroup experienced a fast and far-reaching range expansion during the post-glacial recolonization and is currently spread over the largest area of *A. torrentium* distribution in Europe (Fig. 1), so consequently this phylogroup shows numerous haplotypes separated by small number of mutational steps (Fig. 2c).

Ranges of pairwise patristic distances found between several phylogroups were equal to, or exceeded the typical crustacean species level distinction value of 0.16 substitutions per site, which point to the existence of cryptic species (Additional file 3). However, our *COI* patristic distances between phylogroups are much lower compared to the ranges of patristic distances found for other cryptic crustacean species that represent deep and old divergent lineages [52–54]. We may conclude that the phylogroups within *A. torrentium* are highly divergent but “young” in evolutionary terms, and in a shifting phase from genetic lineages to species, where additional studies of meristic characteristics and their high intra-phylogroup variation (Additional file 7) failed to provide distinct morphological characters that would unambiguously distinguish genetic lineages into species, or that would, at least, further advocate specific status of those highly divergent genetic lineages. To avoid taxonomic inflation, results that indicate an incongruence between morphologic and genetic data should be considered carefully during delimitation of species, whilst leaving the possibility of cryptic species and/or subspecies being in existence [55]. Contrary to this reasoning, Pârvulescu [28] recently described a new species *Austrobotamobius bihariensis*. If accepted as a new species and considering its position in the phylogenetic tree that is not basal, *A. torrentium* would become paraphyletic.

It is difficult to find a suitable nuclear marker with enough resolution to delimit closely related species amongst others because of slower evolution rate of noncoding nuDNA as previously observed for many different species [56, 57]. In the present study the nuclear *ITS2* marker was found suitable for inferring *A. torrentium* phylogenetic tree. Phylogenies inferred from single nuclear genes often have low resolution and low statistical support of the clades [58], but we achieved better resolution by including gaps through simple indel coding method [59] to render the indels phylogenetic information for these tree search methods. We identified lineages recognised also by mtDNA (GK, ZV, APU, LD, ŽPB, KOR, BAN), except for CSE and SB phylogroups that clustered together (Fig. 2b). This clustering was expected since CSE and SB share close evolutionary history [3, 4]. The relationship among phylogroups was unresolved probably due to the lower genetic variability and slower evolutionary rate of *ITS2*, also demonstrated by low intraspecific genetic distances (Additional file 3). The findings agree with other studies that evaluated the diversity of this nuclear gene in crustaceans [47, 60–62]. Obtained values of genetic distances within and between phylogroups were of intraspecific level compared to the interspecific distances found for other European Astacidae (e.g., 1–5% between sister species *Pontastacus* (*Astacus) pachypus* and *Pontastacus (Astacus) leptodactylus* vs. 0.00–0.79% between phylogroup pairs in this study) [47].

The accumulation of characters that contribute to high genetic diversity and intricate phylogeographic patterns are a consequence of numerous events such as vicariant processes and isolation. This is especially pronounced in organisms of limited dispersal potential such as crayfish [63]. Furthermore, such setups are frequently found in organisms distributed in the karst habitats known for their complex and fragmented (paleo)hydrography [54, 64, 65]. One such region is the Dinaric Karst that possesses a high level of biodiversity, with many endemic species of freshwater surface and subterranean fauna [61, 64, 66–68]. A similar effect is observed in the karstic Apuseni Mountains, which represented a refugium that preserved some endemic and relic species of Gastropoda, Isopoda and Diplopoda species [69].

### Evolutionary history

It has been shown that southern Europe and the Balkan Peninsula are regions possessing high plant and animal genetic diversity and are recognised as European biodiversity hotspots [70]. Previous studies of *Austropotamobius torrentium* [3, 4, 27] revealed that its complex evolution was formed from Miocene, to Pleistocene. Distinct evolutionary phylogroups emerged through the intensification of Neotectonic movements and the development of karstification that has a heavily fragmented palaeohydrography, along with periodic climatic shifts during the Pleistocene [3, 4]. Recently, a different perspective on the evolutionary history of *A. torrentium* was proposed [5]. Namely, a new calibration point for species divergence time estimates was used: the separation of the Tisza–Dacia Mega-Unit from the Dinarides that was dated to ~ 16 Ma [5]. According to the authors, this process included “the Tisza–Dacia Mega-Unit (which includes the Apuseni Mountains), which broke away from a larger plate that included the Dinarides and traveled toward the northeast during the Miocene”. Apparently, the process caused the split of the APU phylogroup ancestor, trapped on the “floating island”, from the rest of *A. torrentium*. Reconnection of the Apuseni Mountains freshwater system with other freshwater systems in the area occured ~ 5 Ma [5]. This approach yielded much earlier separation dates for *A. torrentium* and its sister species *A. pallipes*, ~ 42 Ma (HPD 32–54 Ma), as well as the split between *Astacus* and *Austropotamobius* ~ 48.8 Ma (HPD 62.4–37.5 Ma), and among all mtDNA phylogroups of *A. torrentium*, compared to previous estimations. Although Pârvulescu et al. [5] brought a new perspective to the geological history of the *A. torrentium* species complex, it lacked congruence with previous research and molecular clock calibrations [3, 4, 61]. Furthermore, contemporary geological literature indicates an ongoing debate about the geodynamic evolution of the Apuseni Mountains during the Neogene [71 and references within]. Recent integrative studies [72 and references within] point to Paleozoic origin of the Apuseni Mountains that were shaped during Mesozoic and strongly influenced by the contact between Tisza and Dacia Mega-Units during Triasic and Early Jurassic, what indicate the dissconection of the Apuseni and the Dinarids since the Triassic period. Shifts in the region that occurred during the Miocene were primarily related to the deformation and bending of the Eastern Carpathians, and not to the tectonic separation of the Apuseni Mountains from the Dinarides [73]. Until the beginning of the Pliocene, there was a continuous sea or brackish lake between the two areas, while continental conditions with the freshwater lake system began at about 4.5 Ma [74]. Keeping in mind this data, the present study attempted to reconcile both geological calibration approaches and bring a new plausible perspective on *A. torrentium* evolutionary history.

The uplift of Dinarides caused the genus *Austropotamobius* to split into *A. pallipes* to the west of Dinarides, and *A. torrentium* on the east [3, 4, 61]. The uplift of the Dinaric and Carpathian Mountains [75] triggered the isolation of the Pannonian basin from the rest of the Paratethys and the formation of the large brackish/ freshwater Lake Pannon [76]. The complete isolation of the Lake Pannon from the inflow of saline water was estimated to ~ 11.7 Ma [77–79] which coincides with the emergence of the paleo-Danube, discharging directly into the Lake Pannon through its large delta [76, 78]. This caused a change in the depth and water salinity of Lake Pannon, turning it into a shallow brackish/freshwater environment [80]. Together with its northern tributaries, such as the paleo-Tisza, the paleo-Danube formed a shelf margin that prograde from the northwest to the southeast [78]. Klobučar et al. [4] assumed that during this period (probably until ~ 6.5 Ma) the populations of *A. torrentium* in the NCD region were isolated from the east by the large, mostly brackish Lake Pannon and, from the north and west, by mountain ridges of uplifting Dinarides and Alps. Crayfish could survive only in the shallow parts of the lake due to the strong freshwater influx from surrounding rivers. Freshwater conditions are corroborated by findings of freshwater molluscs that were widespread in the shallow parts of the lake ~ 4.5 Ma [74, 76]. Magyar et al. [76] also observed that the paleo-Danube delta lobes in the central part of the Pannonian Basin approached the lower flow of the paleo-Tisza River. This was later confirmed as the shelf margins of the paleo-Danube and the paleo-Tisza were observed as coalesced, and their original, almost perpendicular strike, can be detected until 5.3 Ma [78]. We argue that the connection between the paleo-Danube and Paleo-Tisza Rivers could have allowed the ancestor of the current APU phylogroup to colonise the Apuseni Mountains around 5.3 Ma (Fig. 3).

The paleo-Tisza–paleo-Danube connection coincides with the end of the Messinian Salinity Crisis (MSC) that lasted from ~ 5.96 Ma until ~ 5.33 Ma [81, 82]. The MSC, besides having a strong influence on hydrology, caused increased temperature, aridity and evaporation in the Northern Hemisphere [83]. It is also speculated that the MSC caused a lowering of the water level of the Lake Pannon, at least in its northern part [84]. Thus, *A. torrentium* colonisation of the Apuseni Mountains would be possible at the end of the MSC (~ 5.3 Ma), throughout the northern margin of Lake Pannon (Fig. 1), which is indicated by the lowest genetic distances between ZV/GK phylogroups and APU phylogroup, previously also observed by Pârvulescu et al. [5], and confirmed in this study. Also, during MSC, the sea-level dropped for 50–200 m in the Dacian Basin connected to the Black Sea, situated to the east from Lake Pannon [85]. It is assumed that during the MSC, paleo-Danube ran across the south Carpathians and overflowed from the freshwater Pannonian into the saline Dacian Basin [86]. Therefore, we consider that the northern dispersal route of *A. torrentium* towards the Apuseni region is equally, if not more likely than the previously proposed scenario [5]. It is possible that numerous populations existed in the northern areas, and on the northern dispersal route, but did not survive the adverse climatic conditions during glaciations unlike populations in Apuseni that survived in refugia in karst, similar to the NCD populations. The remnant populations exhibited limited or non post-glacial range expansion and contact indicating the existence of multiple ‘refugia within refugia’ [87], as previously suggested by Klobučar et al. [4].

Formation of the Danube River basin and its drainage network, as we know it today, with its right-sided tributaries (e.g., Velika Morava and Sava), is estimated to Pliocene [78, 88–90]. This, along with the cold climatic conditions [91–93] which are favourable for *A. torrentium* [2] could have allowed its south-eastward spreading. Estimated divergence times between BAN (the most eastern NCD phylogroup) and SB + CSE coincide with this period (Fig. 3 and Additional file 4), which also indicates their closer genetic relatedness compared to other phylogroups (Figs. 1, 2). Further, our results indicate that the divergence between CSE and SB coincide with the beginning of glaciations that started in the Northern Hemisphere during the late Pliocene-early Pleistocene [89, 94] and continued with CSE spreading northward through the Danube River drainage showing a post-glacial leading edge effect as previously suggested in Klobučar et al. [4]. Similar scenarios of post-glacial (re)colonisation of Europe from the southern refugia were recorded for numerous aquatic and terrestrial taxa [51, 95, 96].

### Species delimitation

Molecular species delimitation proved to be a valuable tool for the species identification as a stand-alone method or as part of an integrative taxonomic approach [97]. Contrary to this, a large number of papers reported taxa oversplitting, overlumping or the incongruence among implemented methods [36, 37, 55, 65, 98]. Furthermore, the MOTUs delimited by the analyses of mtDNA represent a hypothesis that should be considered with caution even if well-supported [31]. Species delimitation conducted on our dataset showed a high degree of discordance among methods, with a majority suggesting an unrealistically high number of MOTUs/potential species (9–30) within *A. torrentium* (Fig. 4, Additional file 5). While some of these MOTUs might be the result of revealing previously undescribed diversity, others may be the result of discovering isolated populations currently undergoing speciation [37, 99]. However, in many cases, it is obvious that the analyses oversplit taxa, because the intra-specific genetic divergence for majority of these identified MOTUs is too low to currently consider them as distinct species (Additional file 3). Relatively high genetic divergence (Figs. 2, 5, Additional file 3) indicates that identified MOTUs are in the process of splitting and may evolve into different species in the future [100]. The single locus-based species delimitation approaches, as ABGD, GMYC, bPTP, are known to oversplit taxa and their performance is sensitive to many factors such as higher substitution rates, the number of species included, uneven sampling, varying population sizes, level of gene flow, the number of singletons in the input trees and unresolved nodes [36, 37, 42, 97, 101–105]. Further, species delimitation results inferred on single locus data are known to reflect locus variability, as more variable loci led to a higher number of proposed MOTUs [65]. However, the majority of these delimitations are taxonomically uninformative. Furthermore, most of these methods have been designed for species-rich data sets [32, 106]. Performance of species delimitation approaches can also be affected by the ratio of population sizes to species divergence times [97]. Failure to sample intermediate haplotypes could also be the reason causing the oversplit in the phylogroup CSE, BAN and especially SB due to incomplete geographical coverage, so further sampling could help resolving this oversplitting scenario. The higher number of MOTUs obtained by the tree-based analyses could be a consequence of the fact that those methods tend to overestimate the number of species and they actually reflect genetic structure of the data showing the population structure within the species [107]. This could be the reason why BFD species delimitation recovered GMYC as the most appropriate model for our data set (Additional file 6), reflecting prominent substructure within *A. torrentium*.

Obtained results again suggested the presence of deep divergence within *A. torrentium*, harbouring monophyletic and geographically isolated phylogroups with their own evolutionary trajectories. It is important to point out that strong divergence is not necessarily dependent on the intrinsic characteristics of a species, but could also represent the landscape dynamics of a species habitat [108]. Dinaric karst with fragmented palaeohydrography created important biogeographical barriers that led to diversification events and strong phylogeographic structure in many taxa on the Balkan Peninsula. Currently observed distribution patterns and diversity of freshwater biota are often connected with the geomorphological features of this region and its geo-climatic history [70, 109, 110].

### Meristics

The meristics ([22], this study Additional file 7) and geometric morphometrics [23] could separate crayfish belonging to different phylogroups to some extent, but variation in studied characters, within groups, was evident. Obtained results demonstrated freshwater crayfish plasticity and high intraspecific morphological variation which reflects both the environmental influence and genetic background. Our research on the morphology of *A. torrentium* has not indicated sufficiently stable diagnostic characters that would be helpful in distinguishing crayfish from different phylogroups. Hence we may conclude that morphological traits are not conserved among phylogenetic lineages. The lack of denticulation on the lower edge of antennal scale (antennal exopodite) was pointed out by Pârvulescu [28] as among the most important distinguishing morphological feature to separate newly described *A. bihariensis* from *A. torrentium* belonging to CSE phylogroup and analysed in his study. Contrary to this, in our study of the largest data set analysed so far and including crayfish from all phylogroups but APU, we found this character variable; as the absence of denticulation was observed in all phylogroups (Additional file 7). Accordingly, neither can we use this character, nor any other tested characters reliably in the description of a new species. At the moment, based on the obtained results, we may conclude that observed mtDNA/nuDNA phylogroups present cryptic subspecies [111–114] that should be treated as separate ESUs and, especially ones belonging to the NCD region, should have conservation priority.

### Conservation

Our multigene phylogenetic analyses as well as species delimitation methods revealed that the genetic diversity and evolutionary history of *A. torrentium* is complex and intricate with an everlasting need for further studying (Figs. 1, 2, 3). The geoclimatic processes have left distinguishing signatures in the current distribution and genetics of *A. torrentium* giving rise to highly divergent phylogroups with their own independent evolution. Discovered phylogroups play a fundamental role in the long-term survival and evolution dynamics of *A. torrentium*. Considering that *A. torrentium* shows a decreasing population trend and is listed as vulnerable species in Croatia [13], one of the most important aims of our study was to provide a baseline for the conservation and management of unique genetic variability found within this species through the identification of evolutionary significant units (ESUs). Recognition of ESUs facilitates conservation planning and management without the necessity of formally naming new species or elevating taxa to species level [63]. Taxonomic revision with the description of new species must be a thoughtful process, which considers the whole genus *Austropotamobius* and not only the taxons/groups within *A. torrentium* species-complex*,* so the number of species would not be over- or underestimated. Due to the incongruence between implemented approaches, including lack of morphological characters associated with phylogroups that would be conserved among them (Additional file 7), we were conservative in the inferences drawn from the analyses, and declared phylogroups recovered both on mitochondrial and nuclear DNA as cryptic subspecies and distinct ESUs (ESU1 = BAN, ESU2 = CSE, ESU3 = GK, ESU4 = KOR, ESU5 = LD, ESU6 = SB, ESU7 = ZV, ESU8 = ŽPB, ESU9 = APU) (Additional file 8).

Geographically and genetically isolated phylogroups represent the evolutionary legacy of *A. torrentium* which is highly relevant for conservation due to their mostly small distribution ranges and evolutionary distinctness. Since human mediated translocation and restocking of crayfish for repopulation are encouraged with the aim of increasing the genetic diversity of endangered populations [115], future conservation programs should consider conducting translocations and repopulations only within the same ESU [46, 116–118].

Furthermore, one of the fundamental issues in the conservation of freshwater species is in maintaining genetic diversity by defining the degree of connectivity between populations [119] and finding a balance between outbreeding and inbreeding depression that represent potential threat while restocking/repopulating, so future research should be focused on the study of the genetic structure of phylogroups. Population genetic analyses based on microsatellites can contribute to the understanding of the degree of genetic variation within and among populations, potentially identify management units (MUs) and source populations for future introductions, as well as to reveal recent evolutionary changes and possible population-level hybridisation events through secondary contacts [46, 50, 117, 118, 120]. In addition, cytogenetic research, next generation sequencing and genomic approaches may advance understanding of phylogenetic relationships and taxonomic status of mt and nuDNA phylogroups which, without doubt, play a pivotal role in long term future evolution of *A. torrentium*.

## Conclusions

Results corroborate high genetic diversity within *A. torrentium* preserved in divergent phylogenetic groups.

Because there was no congruence between implemented species delimitation approaches, and we lack establishing morphological characters conserved within lineages, we conclude that established phylogroups, recovered both on mitochondrial and nuclear DNA, are cryptic subspecies and distinct evolutionary significant units that present evolutionary legacy of *A. torrentium* and are highly relevant for conservation due to their mostly small distribution ranges and evolutionary distinctness.

## Methods

To accomplish our aims, we applied a multi-gene molecular approach in the phylogenetic reconstructions and several methods of species delimitation analyses, as well as divergence time estimates using both molecular evolutionary rates and geological/hydrological calibration.

### Sampling, DNA extraction, gene amplification and sequencing

Total of 279 crayfish from 63 locations from Croatia, Slovenia and Republic of North Macedonia were sampled and analysed (Fig. 1, Additional file 1). One pereiopod from each individual was sampled and stored in 96% ethanol at 4 °C until DNA isolation. Sampling was conducted in accordance with ethical standards and all required permissions were obtained from Ministry of Environmental Protection and Energy of the Republic of Croatia. The specimen collections in Slovenia and Republic of North Macedonia were conducted with permissions of local authorities.

Genomic DNA was extracted from muscle tissue using GenElute Mammalian Genomic DNA Miniprep kit (Sigma-Aldrich, St. Louis, MO) following the manufacturer’s protocol, and stored in a freezer until PCR. Mitochondrial *COI* and *16S* rRNA, and nuclear *ITS2* genes were amplified and sequenced, with details provided in the Additional file 9.

### Sequence data and phylogenetic analyses

Sequences were edited using SEQUENCHER 5.4.6 (Gene Codes Corporation, Ann Arbor, MI USA) and aligned using MAFFT [121]. The chromatograms were checked manually for base pair ambiguities and indications for nuclear–mitochondrial pseudogenes (numts) as recommended by Buhay [122]. The *COI* alignment did not contain any length variants or ambiguous sites, while the sequences of the *16S* rRNA cointained length variation. The *ITS2* region contained length variations and nine ambiguous sites. The final alignments were 582 and 476 bp long for *COI* and *16S* rRNA, respectively, while *ITS2* region was 1102 bp long. Number of haplotypes, number of polymorphic sites, and number of parsimony informative sites for each gene alignment was calculated using MEGA X [123] and DnaSP 6.12.03 [124].

The phylogenetic analyses encompassed a total of 1114 *16S* rRNA and *COI* genes sequences of which 642 mtDNA sequences (431 *COI* and 211 *16S* rRNA) were downloaded from GenBank, and 472 sequences (198 *COI* and 274 *16S*) were obtained in this study (Additional file 1). The sequences were collapsed to unique haplotypes with DnaSP 6.12.03 [124]. New haplotypes from this study were deposited in the GenBank and will be publicly available after manuscript acceptance. Phylogenetic analyses were performed on two data sets: the first data set consisted of concatenated *COI* and *16S* rRNA sequences, and the second data set included only *ITS2* sequences. Prior to concatenation, the incongruence length difference test [125] as implemented in PAUP* 4.0a164 [126] was applied to assess congruence between two mitochondrial genes. There was no significant heterogeneity amongst the partitions (P = 0.78), and the final alignment for concatenated mitochondrial sequences was 1058 bp long. *Austropotamobius pallipes* was chosen as an outgroup (GenBank accession numbers for *COI*: KX369673, KX369674; and *16S* rRNA: KX370093, KX370094). Phylogenetic relationships were reconstructed using three different optimality criteria: maximum parsimony (MP), maximum likelihood (ML) and Bayesian analysis (BA), with settings provided in the Additional file 9. Nodes in the phylogenetic trees with bootstrap values P ≥ 75 in ML and MP, and posterior probabilities (pp) values ≥ 0.95 in BA were considered supported.

### Haplotype networks and genetic diversity

Median-joining (MJ) network approach [127] was used to visualise intraspecific evolutionary relationships and haplotype relatedness within *A. torrentium* on concatenated mitochondrial data set using the PopArt [128]. Phylogenetic network using statistical parsimony was constructed for the *COI* gene using the TCS 1.21 software [129] and visualised using tcsBU [130].

Pairwise comparison of uncorrected sequence divergences (p-distances) and corrected Kimura’s two-parameter distances (K2P) between and within phylogroups for *COI*, *16S* rRNA and *ITS2* was performed in MEGA X [123]. The pairwise patristic distances were computed from the ML tree using the program PATRISTIC v1.0 [131] with the aim of comparing obtained values with the proposed crustacean species delimitation threshold of 0.16 substitutions per site in the mitochondrial *COI* gene [39].

### Time of divergence

In order to estimate divergence times among mtDNA phylogroups, concatenated data set (*COI* and *16S* rRNA) was used in the Bayesian statistical framework implemented in BEAST 2.5.2 [132]. The analyses were run on the Cipres Science Gateway [133]. For this purpose, seven different calibration approaches were employed (three molecular and four geological). Molecular clock calibrations were based on the arthropod substitution rate of 2.3% pairwise sequence divergence (0.0115 subs/s/Ma/l) [134, 135], and the decapod substitution rate of 1.4% pairwise sequence divergence (0.007 subs/s/Ma/l) [136] for *COI* partition along with an estimated molecular clock for the *16S* rRNA partition of mtDNA data set. In the third approch, we implemented substitution rates according to Schubart et al. [137] with setting the meanRate prior as a uniform distribution between 0.0083–0.01165 subs/s/Ma/l for *COI* and 0.00325–0.0044 subs/s/Ma/l for *16S* rRNA. Following Klobučar et al. [4] we used mid-points of these intervals (0.0099 for *COI* and 0.0038 for *16S* rRNA) as an ucld.mean prior. For the geological calibrations of the molecular clock, we used three previously described approaches. Firstly, we used the episode of intense uplifting of the Dinarids [138] that caused the split between *A. pallipes* and *A. torrentium* estimated to ~ 12.5 Ma and ~ 16 Ma [for details see [4] and [61]]. TreeModel prior distribution was set to normal, with a mean of 12.5 Ma or 16 Ma and a standard deviation of 0.5. The second approach was based on the tectonic separation of the Apuseni Mountains (Tisza–Dacia microplate) from Dinarides that, according to Pârvulescu et al. [5], took place 16 Ma and it was used as a calibration point for splitting between APU and other NCD phylogroups. TreeModel prior distribution was set to normal, with a mean of 16 Ma and standard deviation of 0.5. For the fourth geological calibration point, we used the occurence of the fluvial connection between the paleo-Danube River and paleo-Tisza River systems that took place around 5.3 Ma [78]. That event could have enabled the colonisation of nowadays north-eastern areal of *A. torrentium* distribution. TreeModel prior distribution was set to normal, with a mean of 5.3 Ma and standard deviation of 0.5. Divergence time estimates were calculated using relaxed molecular clock with log normal distribution, birth–death model of speciation, independent substitution models assigned to mtDNA genes, and run for 150,000,000 generations with details provided in Additional file 9.

### Species delimitation and validation

Application of multiple species delimitation approaches is generally preferable comparing to reliance on a single method [55]. Several methods of single-locus species delimitation were conducted using: the Automatic Barcode Gap Discovery (ABGD) method of Puillandre et al. [101], the General Mixed Yule Coalescent (GMYC, single threshold algorithm) method of Pons et al. [139], the Bayesian implementation of the Poisson Tree Processes (bPTP) method of Zhang et al. [140] and multi-rate Poisson Tree Process (mPTP) method of Kapli et al. [141]. Molecular species delimitation methods generate a certain number of MOTUs and were applied only to the *COI* dataset due to the largest number of available sequences and higher variation levels compering to other markers (e.g. *16S* rRNA and *ITS2*).

The ABGD, genetic pairwise distances based method, was performed using the online version of the program [101] with default parameters and Kimura 2 parameter (K2P) model. Tree-based methods, such as GMYC, bPTP and mPTP, employ a phylogenetic tree as input for the analysis. The GMYC method was performed using the time-calibrated ultrametric tree based on *COI* gene obtained using BEAST 2.5.2, and was run using the SPLITS package [142] in R. The same input tree was used for both bPTP and mPTP methods [140, 141]. The details regarding reconstruction of input tree for species delimitation analyses are reported in the supplementary data (Additional file 9). Boundaries of potential species were also inferred by using the statistical parsimony network reconstruction software TCS [129].

We estimated *A. torrentium* single-locus species trees using *BEAST v.2.5.2 [143] with the same parameters as for species delimitation. The *COI* haplotypes were assigned into different species trees topologies according to the results of phylogeny and species delimitation analyses (ABGD lumper and splitter partitions strategy—in the further text ABGD lumper and splitter [144], TCS, GMYC, bPTP, mPTP), as well as the assumption that all crayfish belong to the same species. Bayes factor delimitation (BFD) approach was applied to compare candidate *BEAST species tree models based on Bayes factors (BF) [145]. Nested sampling analysis [146] was used for the marginal likelihood estimation (MLE) of each species tree [147] in order to calculate the BFs between two models, with details in Additional file 9. The multi-locus species tree was estimated using *BEAST on data set comprising three loci (*COI*, *16S* rRNA, *ITS2*) sampled from 38 individuals representing nine phylogroups of the stone crayfish. We imported three aligments along with additional file with recoded gaps as matrix of binary characters. *BEAST co-estimated three gene trees embedded in a shared species tree and the analysis was run for 150,000,000 generations using the birth–death tree prior and a relaxed molecular clock with an uncorrelated log-normal distribution. Previously established substitution models were assigned to each datasets, with *A. pallipes* as outgroup. The substitution rate for *COI* and *16S* rRNA were set according to Schubart et al. [138] and estimated rate for *ITS2*. Gene trees for mitochondrial genes were linked, while nuclear unlinked. Species tree was visualised in DensiTree v.2.2.6 [199].

### Meristics

Meristic characteristics were examined under a magnifying glass by the same researcher. In total, 749 crayfish collected during the period of the last 20 years, were examinded and 735 were included into analyses, covering all phylogroups except APU that was previously analysed by Pârvulescu [28] (Additional file 1, Additional file 9).

We recorded: number of spines on the ventral side of the merus of the third maxilliped, presence and pronunciation of rostral crista, and absence/presence and type of denticulation (spines or tubercles) on the lower surface of the antennal exopod. Additionaly, in males, shape of the tip of the endopodit of the first and the second gonopod, and the length of the exopodit of the second gonopod were noted. All bilateral characters were recorded for the right side of the body, because previous studies showed that there are no significant differences in their distribution on the two body sides [22]. All details on studied meristic characteristics are given in, Additional file 7, Additional file 9.

Differences in the recorded meristic characters (ordinal variables) between phylogroups were tested by nonparametric Kruskal–Wallis ANOVA and chi-square test in STATISTICA 13.5.

## Supplementary information

**Additional file 1:** List of *Austropotamobius torrentium*, as well as *Austropotamobius pallipes*, *Astacus astacus* and *Pontastacus leptodactylus* sequences used in the analyses. The information comprises the name of the country and sampling site, mtDNA phylogroup, *COI* haplotype ID and their GenBank Accession numbers, *16S* haplotypte ID and their GenBank Accession numbers, concatenated sequences ID, *ITS2* ID and their GenBank Accession numbers and bibliographic references.

**Additional file 2:** TCS phylogenetic network based on *Austropotamobius torrentium COI* gene.

**Additional file 3:** Estimates of evolutionary divergence over sequence pairs of *Austropotamobius torrentium*. Range values of within and between genetic distances of nine *A. torrentium* mtDNA phylogroups (ZV—Zeleni Vir; GK—Gorski Kotar; ŽPB—Žumberak, Plitvice and Bjelolasica; LD—Lika and Dalmatia; BAN—Banovina; SB—southern Balkans; CSE—central and southeastern Europe; APU—Apuseni) for *COI*, *16S* rRNA and *ITS2*. Observed ranges of pairwise patristic distances for *COI* within and between the phylogroups measured on ML tree are also provided.

**Additional file 4:** Estimation of divergence times based on *Austropotamobius torrentium* mitochondrial data set using three molecular clock and four geological calibrations.

**Additional file 5:** Results of species delimitation analyses performed on *Austropotamobius torrentium COI* dataset applying different methods (ABGD (lumper, splitter), TCS, bPTP, mPTP and GMYC).

**Additional file 6:** Results of Bayes factor species delimitation (BFD) based *Austropotamobius torrentium COI* dataset.

**Additional file 7:** Results of *Austropotamobius torrentium* meristic characteristics analyses.

**Additional file 8:** Map of proposed evolutionary significant units (ESUs)/cryptic subspecies for *Austropotamobius torrentium*. The map depicted in figure was produced in ArcGIS 10.3 program package and finished in the program package FreeHand MXa by authors of this study.

**Additional file 9:** Material and methods extended. The detailed information about gene amplification and sequencing, phylogenetic reconstruction, time of divergence estimates, species delimitation and validation, and analyses of meristic characteristics. Additional file also includes list of used references.

## Data Availability

Materials (samples) used in this study are stored in the astacological collection at the Department of Biology, University of Zagreb, while the DNA sequence data supporting the results of this article are available in the GenBank® repository (https://www.ncbi.nlm.nih.gov) under accession numbers (Additional file 1).

## Abbreviations

ABGD: Automatic barcode gap discovery
ANOVA: Analysis of variance
BA: Bayesian analysis
BEAST: Bayesian evolutionary analysis sampling trees
BF: Bayes factors
BFD: Bayes factor delimitation
BIC: Bayesian information criterion
bPTP: Bayesian implementation of the Poisson tree processes
BP: Base pair
BS: Bootstrap
*COI*: Cytochrome *c* oxidase subunit I
CSE: Central and South-eastern Europe
SB: Southern Balkans
BAN: Banovina
GK: Gorski Kotar
LD: Lika and Dalmatia
ZV: Zeleni Vir
ŽPB: ŽUmberak, Plitvice and Bjelolasica
APU: Apuseni Mountain
KOR: Kordun
ESS: Effective sample size
ESU: Evolutionary significant unit
GMYC: General mixed yule coalescent
HKY + G: Hasegawa–Kishino–Yano model with gamma distribution
HKY + I + G: Hasegawa–Kishino–Yano model with invariabe sites and gamma distribution
HKY + F + I + G: Hasegawa–Kishino–Yano model with empirical base frequencies, invariabe sites and gamma distribution
HPD: Highest posterior density
ICS: Indigenous crayfish species
*ITS2*: Second internal transcribed spacer
IUCN: International Union for Conservation of Nature
JC: Jukes–Cantor model
K2P: Kimura 2 parameter model
K3Pu + F + I + G: Kimura 3-parameter with unequal, empirical base frequencies, invariabe sites and gamma distribution
Ma: Million years ago
MCMC: Markov Chain Monte Carlo
MJ: Median-joining
ML: Maximum likelihood
MLE: Marginal likelihood estimation
MMCM: Metropolis-coupled Monte Carlo Markov chains
MOTU: Molecular operational taxonomic unit
MP: Maximum parsimony
mPTP: Multi-rate Poisson tree process
MSC: Messinian salinity crisis
mtDNA: Mitochondrial deoxyribonucleic acid
MU: Management unit
NCD: Northern-central dinarides
nuDNA: Nuclear deoxyribonucleic acid
PCR: Polymerase chain reaction
PP: Posterior probabilities
TCS: Templeton, Crandall and Sing method
