# Supplementary material for: New insights into the genetic diversity of the stone crayfish: taxonomic and conservation implications
Source: BMC Evol Biol. 2020 Nov 6;20:146. doi: 10.1186/s12862-020-01709-1 (PMC7648294; doi:10.1186/s12862-020-01709-1)
Supplement: Supplementary file 2 — Additional file 2: TCS phylogenetic network based on Austropotamobius torrentium COI gene. [file 12862_2020_1709_MOESM2_ESM.docx]

**Additional file 2**

TCS phylogenetic network based on *Austropotamobius torrentium* *COI* gene. The size of the circle is proportional to the frequency of the haplotype scaled with the number of localities in which it occurs. Different colours denote different phylogroups (legend is shown in the upper-left corner) and numbers present *COI* haplotypes IDs given in Additional file 1.

.
