# Supplementary material for: New insights into the genetic diversity of the stone crayfish: taxonomic and conservation implications
Source: BMC Evol Biol. 2020 Nov 6;20:146. doi: 10.1186/s12862-020-01709-1 (PMC7648294; doi:10.1186/s12862-020-01709-1)
Supplement: Supplementary file 4 — Additional file 4: Estimation of divergence times based on Austropotamobius torrentium mitochondrial data set using three molecular clock and four geological calibrations. [file 12862_2020_1709_MOESM4_ESM.docx]

**Additional file 4**

Estimation of divergence times based on *Austropotamobius torrentium* mitochondrial data set using three molecular clock and four geological calibrations. Molecular calibrations included: a) arthropod substitution rate of 2.33% pairwise sequence divergence (0.0115 subs/s/Ma/l) [134, 135]; b) substitution rate range (meanRate priors were set between 0.0083–0.01165 subs/s/Ma/l for *COI* and 0.00325–0.0044 subs/s/Ma/l for *16S* rRNA; mid-points of these intervals were 0.0099 for *COI* and 0.0038 for *16S* rRNA) [4, 137]; c) decapod substitution rate of 1.4% pairwise sequence divergence (0.007 subs/s/Ma/l) [136]. Geological calibrations included d) intense uplifting of the Dinarids ~12.5 Ma and e) ~16 Ma [3, 4, 61]; f) tectonic separation of the Apuseni Mountains [5]. Horizontal node bars depict the 95% HPD intervals and are coloured according to posterior probability support (blue bars - posterior probabilities > 0.95; orange bars - posterior probabilities 0.50-0.95, green bars - posterior probabilities < 0.50). Median and 95% HPD values (in brackets) are given in Ma. Abbreviations used for phylogroups: APU - Apuseni; ZV - Zeleni Vir; GK - Gorski Kotar; LD - Lika and Dalmatia; KOR - Kordun; ŽPB - Žumberak, Plitvice and Bjelolasica; BAN - Banovina; SB - southern Balkans; CSE - central and south-eastern Europe.

**
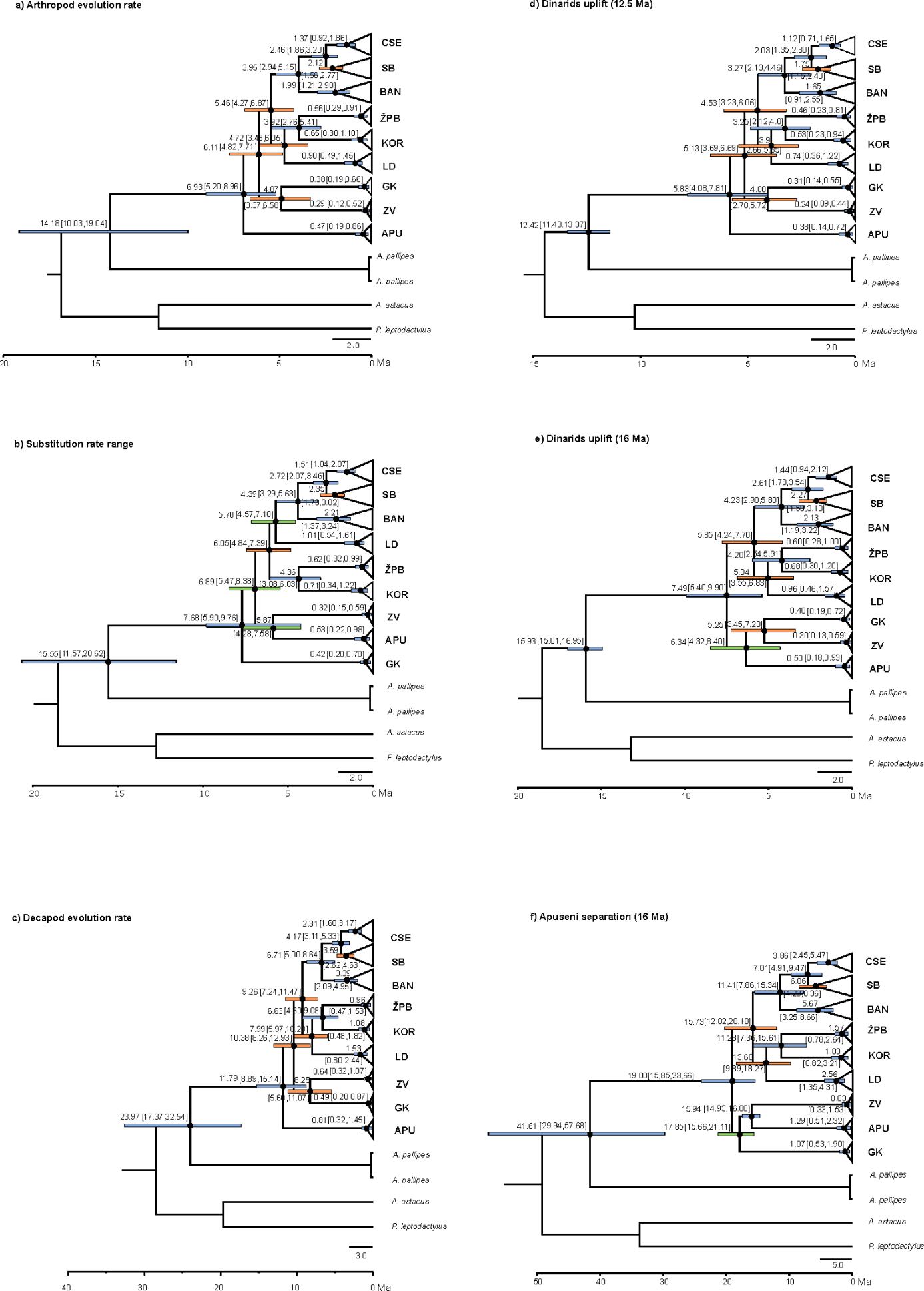
**

Key-events in the *A. torrentium* species evolution based on newly implemented paleo-Tisza - paleo-Danube calibration include following:

(1) split between *A. pallipes* and *A. torrentium* caused by the vicariant effect of the Dinaride orogeny (~13.24 Ma (HPD 18.70-8.55 Ma));

(2) concurrent and rapid divergence of NCD phylogroups restricted to the area between the Lake Pannon and the Dinarides (~6.13 Ma (HPD 8.21-4.64 Ma)) that was facilitated by the intensification of the Dinaric karstification which resulted in heavily fragmented palaeohydrography enhancing allopatric speciation;

(3) colonisation of the Apuseni Mountains throughout the delta systems of the paleo-Danube and paleo-Tisza on the northern shelf margin of the Lake Pannon and splitting from the common ancestor with NCD (~5.10 Ma (HPD 6.07-4.11Ma));

(4) divergence of CSE + SB from a common ancestor with BAN phylogroup enabled by formation of the paleo-Danube drainage system (~3.67 Ma (HPD 5.22-2.46 Ma));

(5) divergence of SB and CSE phylogroups (~2.25 Ma (HPD 3.21-1.51)) followed by postglacial (re)colonization of northern parts of *A. torrentium* distribution range through leading edge expansion of CSE phylogroup during Pleistocene (Fig. 1).
