# Supplementary material for: New insights into the genetic diversity of the stone crayfish: taxonomic and conservation implications
Source: BMC Evol Biol. 2020 Nov 6;20:146. doi: 10.1186/s12862-020-01709-1 (PMC7648294; doi:10.1186/s12862-020-01709-1)
Supplement: Supplementary file 5 — Additional file 5: Results of species delimitation analyses performed on Austropotamobius torrentium COI dataset applying different methods (ABGD (lumper, splitter), TCS, bPTP, mPTP and GMYC). [file 12862_2020_1709_MOESM5_ESM.docx]

**Additional file 5**

Results of species delimitation analyses. Several methods of single-locus species delimitation were conducted using: Automatic Barcode Gap Discovery method (ABGD lumper and splitter approach), TCS method, Bayesian implementation of the Poisson Tree Processes method (bPTP with Bayesian support values (BS) shown in brackets), multi-rate Poisson Tree Process method (mPTP) and General Mixed Yule Coalescent method (GMYC, single threshold algorithm) with node support value shown in brackets (NSV). The first two columns represent phylogroups as in Klobučar et al. [4] and Pârvulescu et al. [5]. In the last column corresponding *COI* haplotypes ID are given (Details in Additional file 1). Abbreviations used for phylogroups: ZV - Zeleni Vir; GK - Gorski Kotar; ŽPB - Žumberak, Plitvice and Bjelolasica; LD - Lika and Dalmatia; BAN - Banovina; SB - southern Balkans; CSE - central and south-eastern Europe; APU - Apuseni.

| **Phylogroups as in** [4] | **Phylogroups as in** [5] | **ABGD lumper** | **ABGD splitter** | **TCS** | **bPTP (BS)** | **mPTP** | **GMYC (NSV)** | **Haplotypes** |
| --- | --- | --- | --- | --- | --- | --- | --- | --- |
| CSE | CSE | G1 | G1 | G1 | S24 (0.44) | S1 | S12 (1) | 150, 152, 153 |
|  |  |  |  |  |  |  | S11 (0.24) | 95, 5, 66, 151, 16, 17, 65, 6 |
|  |  |  |  |  |  |  | S9 (0.12) | 141, 140, 11, 10, 22, 142, 139, 13, 123, 126, 127, 72, 8, 75, 9, 112, 76, 107, 109, 111, 74, 108, 122, 110, 71, 125, 14, 70, 113, 7, 69, 68, 119, 67, 143, 15, 144, 114, 73, 128 |
|  |  |  |  |  |  |  | S10 (0.49) | 12, 124, 145, 146, 147, 18, 19, 20, 21, 23 |
|  |  |  |  |  | S18 (0.99) | S2 | S25 (n/a) | 88 |
|  |  |  |  |  | S17 (0.55) |  | S13 (0.53) | 103, 96, 120, 115, 118, 121 |
|  |  |  |  |  | S15 (0.99) |  | S26 (n/a) | 64 |
|  |  |  |  |  | S14 (0.91) | S3 | S14 (1) | 2, 3, 4 |
| SB | SB |  | G2 | G15 | S5 (0.95) | S6 | S2 (1) | 82, 81 |
|  |  |  | G3 | G13 | S10 (0.91) | S4 | S1 (1) | 78, 80, 79 |
|  |  |  | G4 | G10 | S9 (1) | S5 | S23 (n/a) | 1 |
|  |  |  | G5 | G12 | S8 (0.84) | S8 | S3 (1) | 37, 36, 34, 116, 38 |
|  |  |  | G6 | G11 | S16 (1) | S7 | S24 (n/a) | 94 |
|  |  |  |  |  | S20 (0.77) |  | S6 (0.46) | 35 |
|  |  |  |  |  | S19 (0.63) |  |  | 40, 33 |
|  |  |  | G7 | G16 | S30 (0.73) | S9 | S5 (0.85) | 83 |
|  |  |  |  |  | S29 (0.73) |  |  | 32 |
|  |  |  | G8 | G17 | S22 (0.83) | S10 | S4 (0.41) | 84 |
|  |  |  |  |  | S21 (0.73) |  |  | 117, 137 |
|  |  |  | G9 | G18 | S6 (0.95) | S11 | S7 (1) | 39, 63 |
|  |  |  | G10 | G14 | S7 (0.83) | S12 | S8 (1) | 85, 77 |
| BAN | BAN | G2 | G11 | G2 | S3 (0.91) | S13 | S15 (0.97) | 130, 129, 92, 131, 97, 132, 99, 93, 134, 133 |
|  |  | G9 | G12 | G3 | S (1) | S14 | S27 (n/a) | 41 |
| ŽPB | ŽPB | G5 | G15 | G9 | S28 (0.67) | S18 | S17 (0.87) | 47, 51, 48, 46 |
|  |  |  |  |  | S27 (0.60) |  | S16 (0.52) | 44, 49, 45, 138, 105, 43, 104, 42, 50, 100 |
| LD | LD | G4 | G14 | G8 | S11 (1) | S16 | S28 (n/a) | 60 |
|  |  |  |  |  | S23 (0.49) | S15 | S18 (0.44) | 58 |
|  |  |  |  |  |  |  |  | 62, 61, 59, 89, 91, 90, 57 |
| n/a | n/a | G7 | G17 | G7 | S12 (0.87) | S17 | S19 (0.97) | 98, 102 |
|  |  |  |  |  | S13 (0.99) |  | S29 (0.03) | 106 |
| ZV | ZV | G6 | G16 | G6 | S2 (0.69) | S20 | S21 (0.88) | 52, 53, 54, 55, 56 |
| n/a | APU | G8 | G18 | G5 | S25 (0.93) | S21 | S22 (0.38) | 148 |
|  |  |  |  |  | S26 (0.93) |  |  | 149 |
| GK | GK | G3 | G13 | G4 | S1 (0.73) | S19 | S20 (0.3) | 87, 28, 27, 136, 86, 26, 135, 101, 31, 29, 30, 25, 24 |
