# Supplementary material for: New insights into the genetic diversity of the stone crayfish: taxonomic and conservation implications
Source: BMC Evol Biol. 2020 Nov 6;20:146. doi: 10.1186/s12862-020-01709-1 (PMC7648294; doi:10.1186/s12862-020-01709-1)
Supplement: Supplementary file 6 — Additional file 6: Results of Bayes factor species delimitation (BFD) based Austropotamobius torrentium COI dataset. [file 12862_2020_1709_MOESM6_ESM.docx]

**Additional file 6**

Results of Bayes factor species delimitation (BFD) based *Austropotamobius torrentium* *COI* dataset. Marginal likelihood estimates (MLE) and Bayes factors (2lnBF) were calculated for each species tree model (for details see Additional file 5); mtDNA phylogroups (each phylogroup presents potential species), Automatic Barcode Gap Discovery method - ABGD lumper approach and ABGD splitter approach / TCS (these two methods delimited identical number of potential species), General Mixed Yule Coalescent method - GMYC, Bayesian implementation of the Poisson Tree Processes method - bPTP, multi-rate Poisson Tree Process method - mPTP and *A. torrentium* presents only one species. The model receiving the best MLE score is ranked as first and given in bold (its 2lnBF equals 0).

| **Model** | **MLE** | **SD** | **2lnBF** | **Rank** |
| --- | --- | --- | --- | --- |
| mtDNA phylogroups | -4310 | 4.61 | 230 | 5 |
| ABGD lumper | -4357 | 4.87 | 324 | 6 |
| ABGD splitter =TCS | -4289 | 4.46 | 188 | 4 |
| GMYC | **-4195** | **3.90** | **0** | **1** |
| bPTP | -4228 | 4.09 | 66 | 2 |
| mPTP | -4253 | 4.28 | 116 | 3 |
| *A. torrentium* - one species | -4539 | 5.77 | 688 | 7 |
