# Supplementary material for: New insights into the genetic diversity of the stone crayfish: taxonomic and conservation implications
Source: BMC Evol Biol. 2020 Nov 6;20:146. doi: 10.1186/s12862-020-01709-1 (PMC7648294; doi:10.1186/s12862-020-01709-1)
Supplement: Supplementary file 7 — Additional file 7: Results of Austropotamobius torrentium meristic characteristics analyses. [file 12862_2020_1709_MOESM7_ESM.docx]

**Additional file 7**

Number of *Austropotamobius torrentium* specimens (m = male, f = female) used in meristic analyses. Abbreviations used for phylogroups: BAN - Banovina; CSE - central and south-eastern Europe; GK - Gorski Kotar; KOR - Kordun; LD - Lika and Dalmatia; SB - southern Balkans; ZV - Zeleni Vir; ŽPB - Žumberak, Plitvice and Bjelolasica.

| **mtDNA phylogroup** | **Total** | **m** | **f** |
| --- | --- | --- | --- |
| BAN | 63 | 25 | 38 |
| CSE | 201 | 115 | 86 |
| GK | 107 | 63 | 44 |
| KOR | 9 | 4 | 5 |
| LD | 111 | 75 | 36 |
| SB | 92 | 49 | 43 |
| ZV | 44 | 13 | 31 |
| ŽPB | 108 | 70 | 38 |
| total | 735 | 414 | 321 |

Statistically significant differences (given in bold) between phylogroups in the number of spines on the merus of the third maxilliped.

| phylogroup | \| BAN \| \| --- \| | \| CSE \| \| --- \| | \| GK \| \| --- \| | \| KOR \| \| --- \| | \| LD \| \| --- \| | \| SB \| \| --- \| | \| ZV \| \| --- \| |
| --- | --- | --- | --- | --- | --- | --- | --- | --- | --- | --- | --- | --- | --- | --- |
| \| CSE \| \| --- \| | 1.00 |  |  |  |  |  |  |
| \| GK \| \| --- \| | 1.00 | 1.00 |  |  |  |  |  |
| \| KOR \| \| --- \| | 1.00 | 1.00 | 1.00 |  |  |  |  |
| \| LD \| \| --- \| | **0.002** | 0.11 | 0.11 | 0.35 |  |  |  |
| \| SB \| \| --- \| | 0.06 | 1.00 | 1.00 | 0.99 | 1.00 |  |  |
| \| ZV \| \| --- \| | **< 0.001** | **< 0.001** | **< 0.001** | 1.00 | **< 0.001** | **< 0.001** |  |
| \| ŽPB \| \| --- \| | 1.00 | **0.03** | 0.30 | 1.00 | **< 0.001** | **< 0.001** | **< 0.001** |


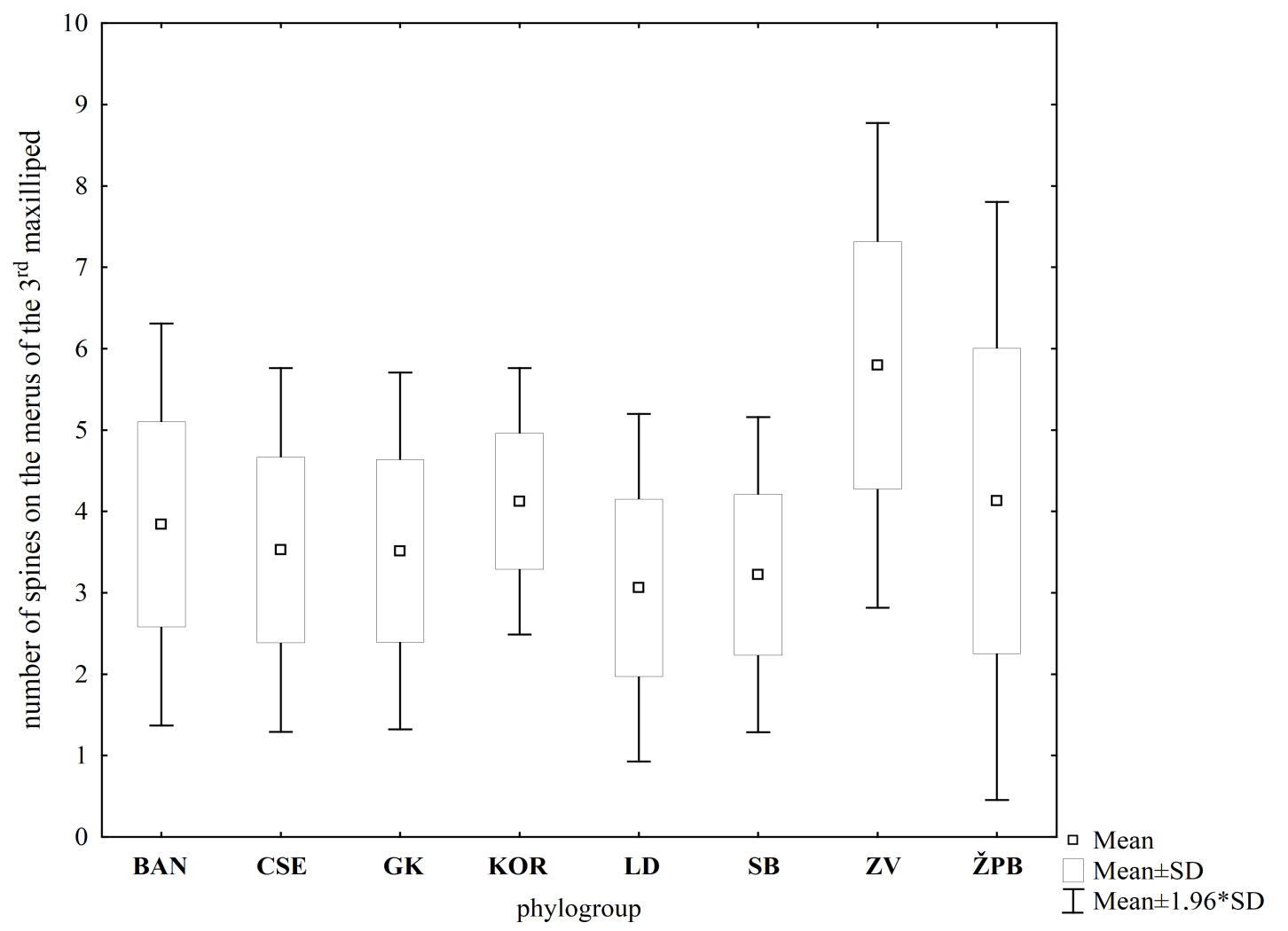


Mean number of spines on the merus of the third maxilliped recorded per *Austropotamobius torrentium* phylogroup .

Percentage of different pronunciation of median rostral crista per *Austropotamobius torrentium* phylogroup*.*

Percentage of different type of pronunciation on the the antennal exopodite per *Austropotamobius torrentium* phylogroup*.*

Percentage of *Austropotamobius torrentium* males with different length of the tip of the first gonopod per phylogroup.

Percentage of *Austropotamobius torrentium* males with different length of the tip of the second gonopod per phylogroup.

Percentage of *Austropotamobius torrentium* males with different length of the exopodite of the second gonopod per phylogroup.
