# Supplementary material for: New insights into the genetic diversity of the stone crayfish: taxonomic and conservation implications
Source: BMC Evol Biol. 2020 Nov 6;20:146. doi: 10.1186/s12862-020-01709-1 (PMC7648294; doi:10.1186/s12862-020-01709-1)

**Additional file 8**

Map of proposed evolutionary significant units (ESUs)/cryptic subspecies of *Austropotamobius torrentium*. BAN - Banovina; CSE - central and south-eastern Europe; GK - Gorski Kotar; KOR - Kordun; LD - Lika and Dalmatia; SB - southern Balkans; ZV - Zeleni Vir; ŽPB - Žumberak, Plitvice and Bjelolasica; APU - Apuseni.


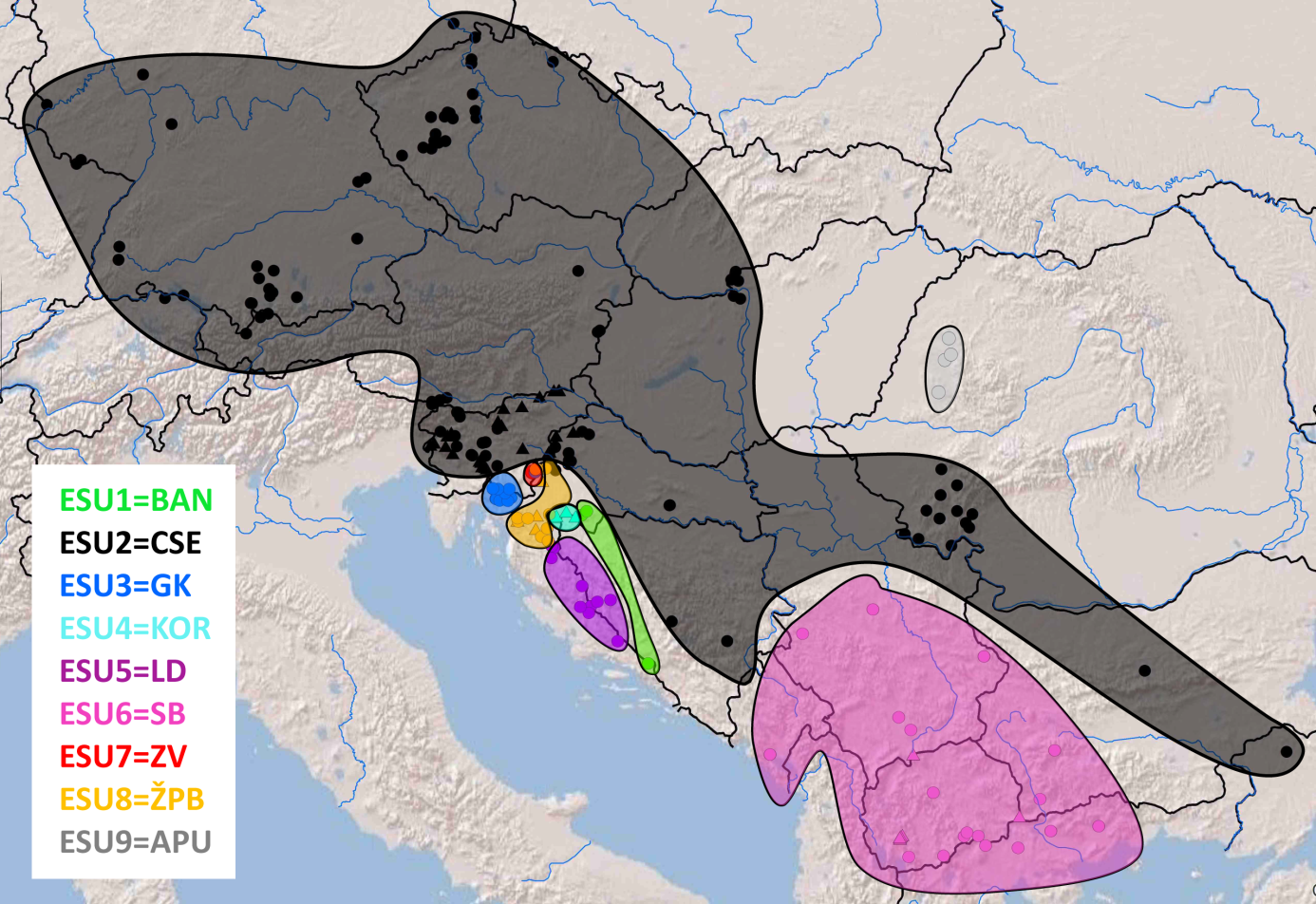

Supplement: Supplementary file 8 — Additional file 8: Map of proposed evolutionary significant units (ESUs)/cryptic subspecies for Austropotamobius torrentium. The map depicted in figure was produced in ArcGIS 10.3 program package and finished in the program package FreeHand MXa by authors of this study. [file 12862_2020_1709_MOESM8_ESM.docx]
