# Supplementary material for: New insights into the genetic diversity of the stone crayfish: taxonomic and conservation implications
Source: BMC Evol Biol. 2020 Nov 6;20:146. doi: 10.1186/s12862-020-01709-1 (PMC7648294; doi:10.1186/s12862-020-01709-1)
Supplement: Supplementary file 9 — Additional file 9: Material and methods extended. The detailed information about gene amplification and sequencing, phylogenetic reconstruction, time of divergence estimates, species delimitation and validation, and analyses of meristic characteristics. Additional file also includes list of used references. [file 12862_2020_1709_MOESM9_ESM.docx]

**Additional file 9**

**Material and methods extended.**

**Gene amplification and sequencing**

The *COI* primer sequences, LCO-1490 and HCO-2198, were adopted from Folmer et al. [1] and the *16S* rRNA primer sequences, 16Sar and 16Sbr were used after Palumbi et al. [2]. The *ITS2* gene was amplified using the primer pair ITSL2 and ITSH1b/m from Schubart et al. [3] and Jelić et al. [61-main refernce list].

The final PCR reaction mix in a total volume of 20 μL for *16S* rRNA gene contained 0.05 U/μL GoTaq G2 HotStart Polymerase, 1.5 mM GoTaq FlexiBuffer, 0.2 mM of each dNTP, 0.275 μmol/μL of each primer, and 10-50 ng/μL of DNA template. The PCR cycling protocol included: initial activation at 95 °C for 3 min, 40 cycles of denaturation at 95 °C for 1 min, annealing at 52 °C for 1 min and extension at 72 °C for 1 min and final extension at 72 °C for 5 min.

The reaction mix in a total volume of 25 μL for *COI* gene contained 0.04 U/μL HotStart Polymerase, 1.5 mM Promega Buffer, 0.15 mM of each dNTP, 0.400 μmol/μL of each primer, 0.7 mM MgCl_2_, and 10-50 ng/μL of DNA template. The PCR cycling protocol included: initial activation at 94 °C for 3 min, 35 cycles of denaturation at 94 °C for 45 s, annealing at 48°C for 1 min and extension at 72 °C for 1 min and final extension at 72 °C for 10 min.

The reaction mix in a total volume of 20 μL for *ITS2* region contained 1.5 mM DreamTaq Mix, 0.400 μmol/μL of each primer, and 10-50 ng/μL of DNA template. The PCR cycling protocol included: initial activation at 95 °C for 3 min, 35 cycles of denaturation at 94 °C for 30 s, annealing at 55 °C for 30 s and extension at 72 °C for 80 min and final extension at 72 °C for 10 min.

The enzymatic purification of PCR products was performed using 2 μL of EXOAnP mix (containing 0.05 U/μL Antarctic Phosphatase and 0.5 U/μL Exonuclease I) for 20 μL of PCR product. The reaction was carried out on a PCR machine for 1 h at 37 °C followed by 20 min at 80 °C.

Sequencing was performed by Macrogen Inc. (Amsterdam, Netherlands) using the same amplification primers.

**Phylogenetic reconstruction**

MP analysis was performed in MEGA X [4] using a tree bisection-reconnection (TBR) algorithm [5] with search level 1 in which the initial trees were obtained by the random addition of sequences (10 replicates). Support for individual clades in MP was evaluated using nonparametric bootstrapping [6] obtained from 10,000 bootstrap replicates. ML analysis was conducted on the IQ-TREE webserver [7]. The optimal model of nucleotide evolution for each partition was calculated using ModelFinder on the IQ-TREE webserver [8, 9]. The selected model for *COI* gene was HKY+F+I+G, while for *16S* rRNA was K3Pu+F+I+G. Branch supports were obtained with the 10,000 bootstrap alignments using ultrafast bootstrap [10] implemented in the IQ-TREE software. The ML tree was inferred using the edge linked partition model [8]. For the BA, the optimal models of nucleotide evolution for each partition of the concatenated data set were selected under the Bayesian information criterion (BIC) using the jModelTest 2.1.10 [11]. The selected model for *COI* was HKY+I+G, and for *16S* rRNA was HKY+G. The BA was performed in MrBayes 3.2.6 [12] with priors set according to the suggested model for each partition. Two separate runs with four Metropolis-coupled Monte Carlo Markov chains (MMCM) were performed for 10,000,000 generations, and trees were sampled every 1,000 generations. After effective sample size (ESS values > 200) for each parameter was confirmed with Tracer v1.7.1 [13], the first 25% of sampled trees were eliminated as burn-in, and a 50% majority-rule consensus tree was constructed, with nodal values representing the posterior probabilities.

Phylogenetic analyses of the *ITS2* region were performed on 72 sequences collapsed into 32 unique haplotypes representing all up till now described mtDNA phylogroups [4, 5 main refernce list] and a new phylogroup discovered in this research. Four *A. pallipes* sequences were used as an outgroup (accession numbers: KX370591, KX370594, KX370597, KX370600). The optimal model of nucleotide evolution was selected under the BIC using jModelTest 2.1.10 [11]. The proposed model was Jukes-Cantor (JC) with equal base frequencies, all substitutions equally likely assessed. Phylogenetic relationships were reconstructed using BA. Prior to BA, all the gaps in the alignment were coded by employing the simple indel coding algorithm as implemented in FastGap [14] that converts gaps to a matrix of binary presence/absence character state, which was implemented in the nexus input file for further analyses. Two separate runs with four MMCM chains were performed for 3,000,000 generations, and trees were sampled every 100 generations. After checking diagnostic parameters of convergence, the first 25% of sampled trees were eliminated as burn-in and a 50% majority-rule consensus tree was constructed, with nodal values representing the posterior probabilities.

All phylogenetic trees were visualised in FigTree v1.4.4 [15].

**Time of divergence**

The molecular clock tests were performed using the ML method in MEGA X by comparing the ML value for the given topology with and without the molecular clock constraints under HKY+G+I (for *COI* data set) and HKY+G (for *16S* rRNA data set) models. The null hypothesis of equal evolutionary rate throughout the tree was rejected at a 5% significance level; therefore, clock model was set as relaxed with an uncorrelated lognormal distribution for all tree calibrations. The likelihood of the Yule versus the birth-death model was estimated with path sampling [21]. The tree prior was set as the birth-death since comparison between Bayes factors (BF) favoured this model over Yule model (2lnBF = 112). Independent substitution models were assigned to each mtDNA gene. Both molecular and geological calibrations were run for 150,000,000 generations. The generated trace files were analysed to check the convergence using Tracer v1.6 [13], 25% of the sampled trees were subsequently discarded as burn-in and the consensus tree was produced in TreeAnnotator v2.5.2 [132-main reference list].

**Species delimitation and validation**

The input tree for GMYC and PTP was constructed using BEAST 2.5.2. with an input file generated in BEAUti as recommended in other studies [16, 17]. BEAST analysis was run for 50,000,000 generations using the using Coalescence tree prior and a relaxed molecular clock with an uncorrelated log-normal distribution. We used the coalescence tree prior because it is considered to be a more adequate option and appears to fit better the majority of the data sets in model comparisons than the Yule prior which results in greater number of entities [16,18]. The substitution model HKY+I+G was assigned to the *COI* datasets and the substitution rate for *COI* was set to 0.0115 subs/s/my/l. Species *Pontastacus leptodactylus, Astacus astacus* and *Austropotamobius pallipes* were used as outgroups, but were later excluded from GMYC analysis using the function *drop.tip( )* from the package APE [19]. After checking the convergence with Tracer, a consensus tree was calculated and used as an input tree for further analyses. The same input tree was used for bPTP and mPTP. The bPTP method was run on the webserver at http://species.h-its.org, while mPTP method was perfomed on the web server at http://mptp.h-its.org/, both using default parameters and outgroups being removed.

Chain length for nested sampling analysis was 200,000, sub-chain length of 10,000 and 20 particles with default value of epsilon (1^-12^). Bayesian factors (2lnBFs) between the two competing models were calculated as suggested by Kass and Raftery [147 – main reference list]. A positive BF (2lnBFs) values reflect evidence in favour of the first model, to which the second model is compared, whereas negative BF values are considered as evidence favouring the second model. Values above 10 (2lnBFs > 10) are considered as decisive evidence in favour of the first model [20].

**Meristics**

Number of spines on the merus of the third maxilliped were counted for each individual, and expressed as numbers per phylogroup. Presence and pronunciation of rostral crista was expressed as absent or present. If rostral crista was present, it was evaluated as one of three “rostrum types”: weak, medium and strong, and were expressed as a percentage of each rostrum type per phylogroup. Denticulation on the antennal exopodite was evaluated as smooth (absence of denticulation/no tubercles or spine) or as with tubercles or spines. Denticulation was expressed as percentage of each denticulation type per phylogroup. Tip of gonopods were recorded/depicted as ratio of tip length to the length of the whole gonopod (1/3 or 1/2 of the gonopod's length); length of the exopodite of the second gonopod was expressed as ratio between exopodite length and the length of the gonopod (1/3 or 1/2 of the gonopod's length). Gonopod “shapes” were expressed as percentage of the “shape” per phylogroup.

**References**

1. Folmer O, Black M, Wr H, Lutz R, Vrijenhoek R. DNA primers for amplification of mitochondrial Cytochrome C oxidase subunit I from diverse metazoan invertebrates. Mol Mar Biol Biotechnol. 1994;3: 294–299.

2. Palumbi SR, Martin A, Romano S, McMillan WO, Stice L, Grabowski G. The Simple Fool’s Guide to PCR (2nd ed.). Honolulu: University of Hawaii; 1991.

3. Schubart CD, Weil T, Stenderup JT, Crandall KA, Santl T. Ongoing phenotypic and genotypic diversification in adaptively radiated freshwater crabs from Jamaica. In Glaubrecht M, editor. Evolution in Action. Berlin, Heidelberg: Springer; 2010. p. 323–349.

4. Kumar S, Stecher G, Li M, Knyaz C, Tamura K. MEGA X: Molecular evolutionary genetics analysis across computing platforms. Mol Biol Evol. 2018:35:1547–1549.

5. Nei M, Kumar S. Molecular evolution and phylogenetics. Oxford: Oxford University Press; 2000.

6. Felsenstein J. Confidence limits on phylogenies: an approach using the bootstrap. Evolution. 1985;39:783–791.

7. Trifinopoulos J, Nguyen LT, von Haeseler A, Minh BQ. W-IQ-TREE: a fast online phylogenetic tool for maximum likelihood analysis. Nucleic Acids Res. 2016;44:232–235.

8. Chernomor O, von Haeseler A, Minh BQ. Terrace aware data structure for phylogenomic inference from supermatrices. Syst Biol. 2016;65:997–1008.

9. Kalyaanamoorthy S, Minh BQ, Wong TK, von Haeseler A, Jermiin LS. ModelFinder: fast model selection for accurate phylogenetic estimates. Nat Methods. 2017;14:587–589.

10. Hoang DT, Chernomor O, von Haeseler A, Minh BQ, Vinh LS. UFBoot2: Improving the Ultrafast Bootstrap Approximation. Mol Biol Evol. 2018;35:518–522.

11. Darriba D, Taboada GL, Doallo R, Posada D. jModelTest2: more models, new heuristics and parallel computing. Nat Methods. 2012;9:772.

12. Ronquist F, Teslenko M, van der Mark P, Ayres DL, Darling A, Höhna S, et al. MrBayes 3.2: efficient Bayesian phylogenetic inference and model choice across a large model space. Syst Biol. 2012;61:539–542.

13. Rambaut A, Drummond AJ, Xie D, Baele G, Suchard MA. Posterior summarisation in Bayesian phylogenetics using Tracer 1.7. Syst Biol. 2018;67:901–904.

14. Borchsenius F. FastGap 1.2. Aarhaus: Department of Biosciences, University Denmark; 2009.

15. Rambaut A. FigTree Version 1.4.4. (Computer Program). Available at http://tree.bio.ed.ac.uk/.

16. Monaghan M, Wild R, Elliot M, Fujisawa T, Balke M, Inward DJ, et al. Accelerated species inventory on Madagascar using coalescent-based models of species delineation. Syst Biol. 2009;58:298–311.

17. Ceccarelli S., Sharkey M, Zaldivar-Riveron A. Species identification in the taxonomically neglected highly diverse neotropical parasitoid wasp genus *Notiospathius* (Braconidae: Doryctinae) based on an integrative molecular and morphological approach. Mol Phylogenet Evol. 2012;62:485–495.

18. Talavera G, Dincǎ V, Vila R. Factors affecting species delimitations with the GMYC model: Insights from a butterfly survey. Methods Ecol Evol. 2013;4:1101–1110.

19. Paradis E, Claude J, Strimmer K.  APE: analyses of phylogenetics and evolution in R. Bioinformatics. 2004;20:289–290.

20. Leaché AD, Fujita MK, Minin VN, Bouckaert RR. Species delimitation using genome-wide SNP Data. Syst Biol. 2014;63:534–542.

21. Condamine F, Nagalingum N, Marshall C, Morlon H. Origin and diversification of living cycads: a cautionary tale on the impact of the branching process prior in Bayesian molecular dating. BMC Evol Biol. 2015;15:65.
